# A subcortical feeding circuit linking an interoceptive node to jaw movement

**DOI:** 10.1038/s41586-024-08098-1

**Published:** 2024-10-23

**Authors:** Christin Kosse, Jessica Ivanov, Zachary Knight, Kyle Pellegrino, Jeffrey Friedman

**Affiliations:** 1 Laboratory of Molecular Genetics, Howard Hughes Medical Institute, The Rockefeller University, New York, NY USA; 2 Department of Physiology, University of California, San Francisco, San Francisco, CA USA

**Keywords:** Hypothalamus, Neural circuits, Sensorimotor processing

## Abstract

The brain processes an array of stimuli, enabling the selection of appropriate behavioural responses, but the neural pathways linking interoceptive inputs to outputs for feeding are poorly understood^1–3^. Here we delineate a subcortical circuit in which brain-derived neurotrophic factor (BDNF)-expressing neurons in the ventromedial hypothalamus (VMH) directly connect interoceptive inputs to motor centres, controlling food consumption and jaw movements. VMH^BDNF^ neuron inhibition increases food intake by gating motor sequences of feeding through projections to premotor areas of the jaw. When food is unavailable, VMH^BDNF^ inhibition elicits consummatory behaviours directed at inanimate objects such as wooden blocks, and inhibition of perimesencephalic trigeminal area (pMe5) projections evokes rhythmic jaw movements. The activity of these neurons is decreased during food consumption and increases when food is in proximity but not consumed. Activity is also increased in obese animals and after leptin treatment. VMH^BDNF^ neurons receive monosynaptic inputs from both agouti-related peptide (AgRP) and proopiomelanocortin neurons in the arcuate nucleus (Arc), and constitutive VMH^BDNF^ activation blocks the orexigenic effect of AgRP activation. These data indicate an Arc → VMH^BDNF^ → pMe5 circuit that senses the energy state of an animal and regulates consummatory behaviours in a state-dependent manner.

## Abstract (web summary)

A subcortical circuit that regulates food consumption in mice is described, involving neurons in the ventromedial hypothalamus that are directly linked to motor centres that regulate feeding and jaw movements.

## Main

The principles of how innate behaviours are generated have long been subject to intense theoretical and experimental inquiry, but the precise neural mechanisms underlying behaviour selection remain largely unknown^1–3^. Feeding is a typical innate behaviour in which an array of sensory and interoceptive inputs are integrated to generate an adaptive behavioural response. However, because these many inputs typically change the probability of feeding, in contrast to a reflex, behaviour initiation is context dependent^4^. Although many brain areas and neuronal types that modulate feeding behaviours have been identified, mapping a complete circuit linking relevant sensory and interoceptive inputs to consummatory behaviours is important for understanding how feeding is regulated. We thus sought to identify a neural pathway that would directly connect interoceptive signalling to premotor centres that control consumption. Identifying the essential components of such a circuit would be informative in its own right and potentially provide a framework for investigating how top-down signals regulate innate behaviours^1^. Here we report the components of a simple circuit regulating feeding that directly connects leptin signalling in the arcuate nucleus (Arc) to the jaw premotor area of the perimesencephalic trigeminal area (pMe5) that controls food consumption.

These elements emerged from studies that aimed to identify the circuit of a population of brain-derived neurotrophic factor (BDNF) neurons that regulate food intake and body weight. A role for BDNF neurons in feeding has been shown in studies of mice and humans carrying mutations in the genes encoding BDNF or its receptor tyrosine kinase B (TrkB)^5–10^. Heterozygous BDNF or TrkB mutations cause overconsumption and massive obesity in animals and humans. In addition, genome-wide association studies^11,12^ have suggested a role for BDNF in the development of obesity. However, the neural circuits responsible for the obesity and hyperphagia seen with defects in BDNF signalling are unknown. As BDNF mutations cause obesity, we initially set out to identify which BDNF neurons normally restrict overfeeding in animals fed a HPD. We found that a discrete subpopulation of BDNF neurons in the ventromedial hypothalamus (VMH) but not elsewhere are activated in diet-induced obese (DIO) mice fed a high-fat diet (HFD). We then aimed to define the functions of these neurons, including identification of their inputs and outputs. Here we report that VMH^BDNF^ neurons act as key nodes regulating food consumption that directly link neurons in the Arc that receive interoceptive inputs to premotor sites in the brainstem that regulate jaw movements and consummatory behaviour.

## VMH^BDNF^ neurons cause obesity

**Fig. 1:**
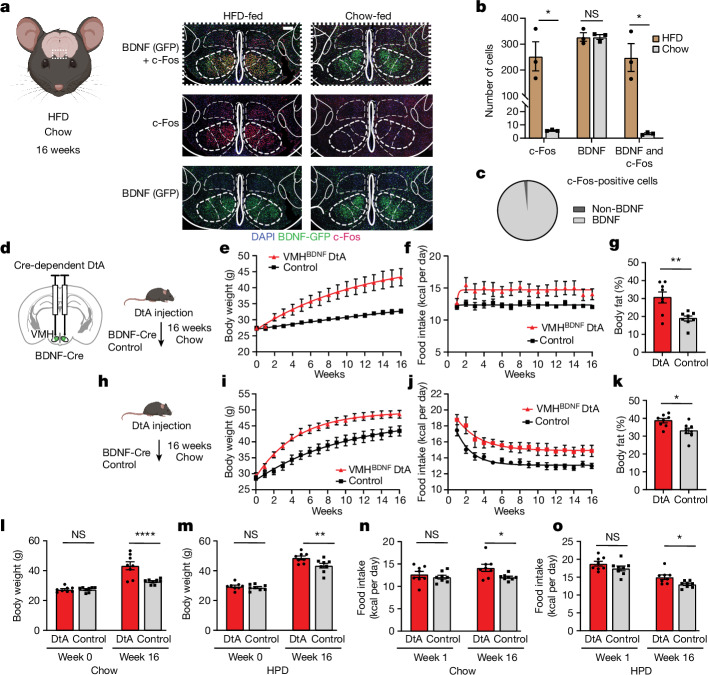
VMH^BDNF^ neurons suppress food intake and body weight. **a**, Schematic of a coronal brain slice containing the VMH and representative example of immunolabelling for c-Fos (red) and BDNF–GFP (green) after 16 weeks of HFD of *n* = 3 mice. **b**, Quantification of c-Fos-positive and BDNF–GFP cells in the VMH of *n* = 3 HFD-fed and *n* = 3 chow-fed mice. **c**, Quantification of c-Fos-positive cells expressing BDNF–GFP after HFD (*n* = 3 mice). **d**, Schematic of bilateral DtA injection and timeline for mice fed chow ad lib. **e**,**f**, Weekly time course of body weights (**e**) and calorie intake (**f**) of chow-fed DtA (*n* = 8) and control (*n* = 8) mice. **g**, Comparison of percentage body fat between DtA (*n* = 8) and control (*n* = 8) mice. **h**, Timeline for mice fed HPD ad lib. **i**,**j**, Weekly time course of body weights (**i**) and calorie intake (**j**) of HPD-fed DtA (*n* = 8) and control (*n* = 8) mice. **k**, Comparison of percentage body fat between DtA (*n* = 8) and control (*n* = 8) mice on HPD. **l**,**m**, Body weight comparison between chow-fed DtA mice (*n* = 8) and control mice (*n* = 8) before DtA ablation and 16 weeks postsurgery (**l**) and HPD-fed mice (**m**) (*n* = 8 mice per group). **n**,**o**, Comparison of average daily calorie intake between chow-fed DtA and control mice at 1 and 16 weeks postsurgery (**n**) and HPD-fed mice (**o**) (*n* = 8 mice per group). In **b**, *t*-tests were performed using the Holm–Sidak method. Unpaired *t*-tests with Welch’s correction were used in **g** and **k**. Two-way repeated-measures analysis of variance (RM ANOVA) with Šídák’s multiple comparisons test was used in **l**–**n**. *P < 0.05, **P < 0.01, ***P < 0.001, ****P < 0.0001. Error bars indicate s.e.m. Scale bar, 200 μm. Elements (mice) in **a**, **d** and **h** were created using BioRender (https://biorender.com). **d** is adapted from ref. ^56^, Elsevier. Source Data

**Extended Data Fig. 1:**
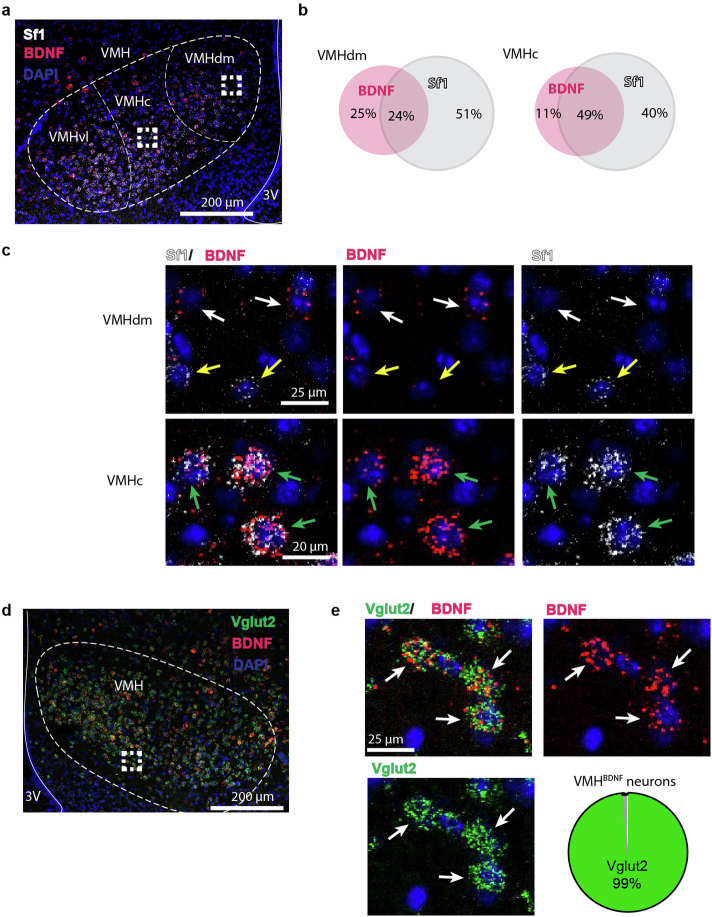
Overlap of VMH^BDNF^ neurons with Vglut2 and Sf1 and effect of TrkB inhibition. **a**, Representative example of coronal sections (n = 3 mice) through the VMH with in situ hybridization for BDNF (magenta) and Sf1 (white) and DAPI (blue). **b**, Quantification of overlap with SF1 and BDNF in dorsomedial and central VMH of n = 3 mice. **c**, High magnification examples of boxed areas from **a** with white arrows indicating BDNF only, yellow arrows Sf1 only, and green arrows BDNF and SF1 expression. **d**, Representative example of coronal sections (n = 3 mice) through the VMH with in situ hybridization for BDNF (magenta) and Vglut2 (green) and DAPI (blue). **e**, High magnification examples of overlap (indicated by white arrows) of boxed areas from **d**. Right, quantification of Glut2 expression in VMH^BDNF^ neurons of 3 mice. *P < 0.05, **P < 0.01, ***P < 0.001, ****P < 0.0001. Error bars ± s.e.m. Source Data

Mutations of the genes encoding BDNF or TrkB, its receptor, cause extreme obesity in mice and humans, indicating that neurons that express these genes normally restrict food intake and weight gain. However, the identity of the BDNF neurons and the underlying neuronal mechanism that regulates feeding in response to body weight changes was unclear. We reasoned that the key subpopulation(s) of BDNF-expressing neurons would be activated when animals were placed on a HFD and gained body weight. This was investigated by identifying BDNF neurons that showed increased expression of c-Fos, an activity marker, in response to a HFD in BDNF–Cre mice mated to Rosa26^fsTRAP^ reporter mice expressing a Cre-dependent GFP. Brain sections from HFD-fed and chow-fed mice were costained for both GFP (BDNF) and c-Fos. This revealed a 50-fold increase in the number of BDNF neurons that expressed c-Fos in the central and dorsomedial regions of the VMH (Fig. 1a,b). Indeed, there was a nearly complete overlap between GFP and c-Fos in VMH, with 98% of the neurons from DIO mice that expressed c-Fos also expressing BDNF (Fig. 1c). The VMH^BDNF^ neurons showed limited overlap with steroidogenic factor 1 (SF1), a canonical marker for VMH, which, as shown below, has different functional effects^13–15^. Furthermore, in situ hybridization of SF1 and BDNF revealed that approximately half of the BDNF neurons in the dorsomedial VMH did not express SF1 (Extended Data Fig. 1a–c), consistent with previous studies using RNA sequencing^16^ and immunohistochemistry^17^ that showed BDNF-expressing neurons to be a distinct subpopulation of the neurons in this nucleus. The data thus suggested that VMH^BDNF^ neurons represent a distinct subpopulation of VMH neurons that are specifically activated in animals fed a HFD. Further experiments (Extended Data Fig. 1d,e) revealed that 99% of the VMH^BDNF^ neurons colocalized with vesicular glutamate transporter 2 (Vglut2) and were thus glutamatergic. To directly test the possibility that these neurons normally restrict food intake and body weight, we analysed the effects of selectively ablating VMH^BDNF^ neurons in mice fed chow or a HFD.

VMH^BDNF^ neurons were ablated by injecting an adeno-associated virus (AAV) expressing a floxed diphtheria toxin A subunit (DtA) construct into the VMH of BDNF–Cre mice (Fig. 1d–o). Chow-fed mice with DtA ablation (Fig. 1d–g) ate significantly more than controls (Fig. 1n), resulting in significantly increased body weight (Fig. 1l) and body fat content (Fig. 1g), consistent with previous results^10^. We next ablated VMH^BDNF^ neurons in animals fed a highly palatable diet (HPD) (Fig. 1h–k). Initially, mice with ablation of VMH^BDNF^ neurons showed overfeeding similar to that of control mice (Fig. 1j,o). However, whereas food intake in control animals fed a HPD gradually decreased over time as weight increased, animals with VMH^BDNF^ neuron ablation continued to consume significantly more food for an extended period. When mice with a VMH^BDNF^ ablation were fed a HFD, their weight ultimately stabilized at a significantly higher level than that of control mice (Fig. 1i,m). Mice with VMH^BDNF^ ablation also showed significantly higher levels of adiposity (Fig. 1k). These results show that VMH^BDNF^ neurons normally restrict overfeeding and obesity in animals fed a HPD or chow diet.

## VMH^BDNF^ neurons regulate food intake

**Fig. 2:**
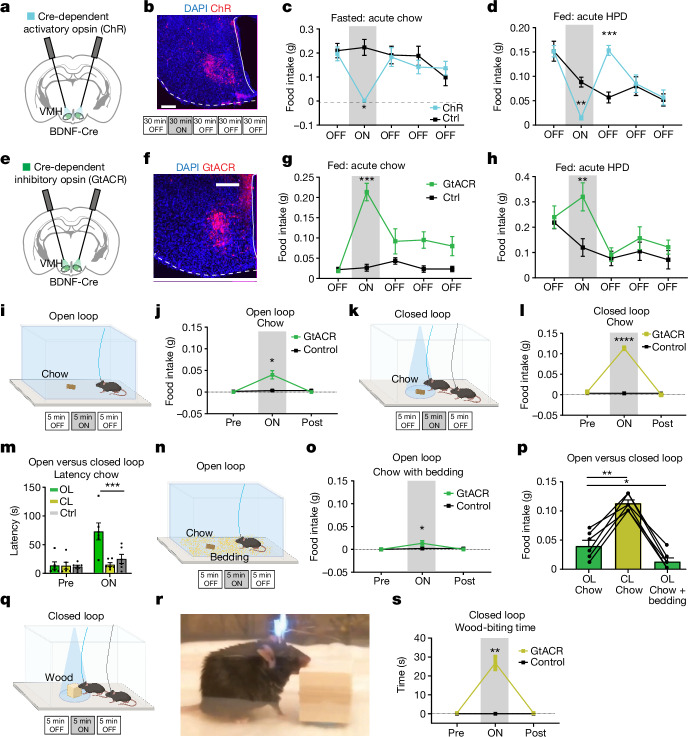
VMH^BDNF^ neuron activity bidirectionally controls food consumption. **a**, Schematic of ChR expression and optic fibre placement. **b**, Example of ChR expression in *n* = 5 mice with experimental timeline. **c**,**d**, Food intake of fasted ChR (*n* = 5) and control (*n* = 5) mice tested with acute chow (**c**) and mice fed ad lib with acute HPD (**d**). **e**, Schematic of GtACR expression and optic fibre placement. **f**, Example of GtACR expression in *n* = 6 mice. **g**,**h**, Food intake of GtACR mice fed chow ad lib (*n* = 6) and control mice (*n* = 6) tested with acute chow (**g**) and with acute HPD (**h**). **i**, Schematic of open loop optogenetic inhibition and experimental timeline. **j**, Quantification of food intake of GtACR (*n* = 6) and control mice (*n* = 6). **k**, Schematic of closed loop optogenetic inhibition and timeline. **l**, Food intake of GtACR (*n* = 6) and control (*n* = 6) mice. **m**, Latency of GtACR mice approaching the chow pellet in open- and closed loop inhibition (*n* = 6). **n**, Schematic of open loop optogenetic inhibition with bedding present and timeline. **o**, Food intake of GtACR (*n* = 6) and control (*n* = 6) mice. **p**, Comparison of food intake during optogenetic inhibition of six GtACR mice in the open loop, closed loop, and open loop with bedding set-up. **q**, Schematic of closed loop optogenetic inhibition with a wood block and experimental timeline. **r**, Example of consummatory biting behaviour displayed upon VMH^BDNF^ neuron inhibition. **s**, Quantification of percentage of time spent biting the wood block for GtACR (*n* = 6) and control (*n* = 6) mice. Two-way RM ANOVA with Šídák’s multiple comparisons test was used in **c**, **d**, **g**, **h**, **j**, **l**, **m**, **o** and **s**. A mixed-effects model (restricted maximum likelihood) with Dunnett’s multiple comparison was used in **p**. *P < 0.05, **P < 0.01, ***P < 0.001, ****P < 0.0001. Error bars indicate s.e.m. Scale bars, 200 µm. Elements in **i**, **k**, **n** and **q** were created using BioRender (https://biorender.com). **a** and **e** are adapted from ref. ^56^, Elsevier. Source Data

To investigate whether VMH^BDNF^ neuron activity regulated food intake, we used optogenetics to either inhibit or activate these neurons. AAV strains encoding channelrhodopsin (ChR) (Fig. 2a–d) or *Guillardia theta* anion ChR (GtACR) (Fig. 2e–h) were injected into the VMH of BDNF–Cre mice, and food intake was measured in response to light. To investigate whether activation of VMH^BDNF^ neurons suppressed feeding, we fasted mice overnight and then presented them with a chow pellet (Fig. 2c). Without light activation, control and ChR mice consumed similar amounts of food during the first 30 min. However, VMH^BDNF^ neuron stimulation at 2 Hz inhibited food intake by 99.8%, and intake returned to control levels when light stimulation ceased. Thus, VMH^BDNF^ neuron activation can entirely suppress the hunger-induced drive to eat. To test whether VMH^BDNF^ neuron activity was also sufficient to suppress hedonic feeding, we presented sated chow-fed mice with a highly palatable pellet with high sugar and high fat content (Fig. 2d). Control and ChR mice consumed similar amounts at baseline, but optogenetic activation of VMH^BDNF^ neurons at 2 Hz significantly decreased intake of the highly palatable pellets by 76%. We also found that in the period after light activation ceased, the mice in which VMH^BDNF^ neurons had been activated consumed even more HPD than controls. These data show that VMH^BDNF^ neural activation significantly diminishes both homeostatic and hedonic feeding.

We next tested the effects of VMH^BDNF^ neuron inhibition by measuring food intake in fed BDNF–Cre animals expressing GtACR in VMH neurons. Whereas the baseline intake of the control and GtACR mice was the same, optogenetic inhibition in the GtACR mice significantly increased food intake (by 1183%) during the 30 min testing period (Fig. 2g). To test whether inhibiting VMH^BDNF^ neuron activity would also increase the intake of HPD, we measured food intake after presenting mice with a HPD pellet. Initial intake before optogenetic inhibition was similar between control and GtACR mice, and light-induced neural inhibition again increased consumption, by 133% (Fig. 2h).

**Extended Data Fig. 2:**
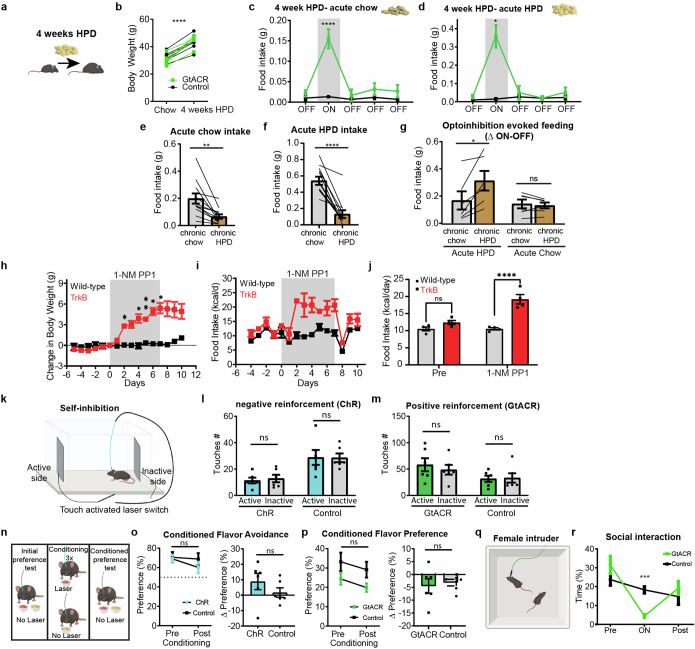
Inhibition of VMH^BDNF^ neurons in the DIO state and VMH^BDNF^ neuron effects on valence and social interaction. **a**, Schematic of a HPD induced obese state. **b**, Changes in body weight after DIO in (n = 6 mice each group) control and GtACR mice. Food intake with optoinhibition in GtACR (n = 6) and control mice (n = 6) both given chow (**c**) and (**d**) HPD after DIO with two-way RM ANOVA with Šídák’s multiple comparisons test. **e**, Cumulative chow intake of all (n = 12) mice before and after DIO during laser off (paired t-test), and **f**, HPD intake (paired t-test). **g**, Optoinhibition evoked feeding of the same mice, two-way RM ANOVA with Šídák’s multiple comparisons test. Time course of body weight (**h**) and food intake of HFD (**i**) with 1-NM PP1 treatments in control (n = 4 each) and TrkB mutant mice and **j**, average food intake before and during 1-NM PP1 treatment (two-way RM ANOVA with Šídák’s multiple comparisons test). **k**, Schematic of touch activated self-inhibition with an active site controlling the laser. Number of touches at active and inactive site for (**l**) ChR (n = 6) and control mice (n = 6) during self-inhibition and (**m**) for positive reinforcement with GtACR (n = 6) and control mice (n = 6) (two-way RM ANOVA with Šídák’s multiple comparisons test). **n**, Schematic of conditioned flavour preference assay. **o**, Preference at the initial test (pre) and conditioned preference test (post) in ChR (n = 6) and control mice (n = 6) (two-way RM ANOVA with Šídák’s multiple comparisons test) and right, change in preference (Welch’s t test). **p**, Left, flavour preference at the initial test (Pre) and conditioned preference test (post) of in GtACR (n = 6) and control mice (n = 6) (two-way RM ANOVA with Šídák’s multiple comparisons test) and right, change in preference (Welch’s t test). **q**, Schematic of social interaction assay. **r**, Time spent in social interactions for GtACR (n = 6) and control mice (n = 6). Two-way RM ANOVA with Šídák’s multiple comparisons test. *P < 0.05, **P < 0.01, ***P < 0.001, ****P < 0.0001. Error bars ± s.e.m. Source Data

We next evaluated our hypothesis that VMH^BDNF^ neurons would be activated by obesity to suppress further weight gain (see data in Fig. 1a–c) by measuring food intake during VMH^BDNF^ inhibition in DIO mice that had been fed a HPD for 4 weeks. Consistent with the studies of VMH^BDNF^ neuron inhibition in lean mice, optogenetic inhibition of these neurons using GtACR in the obese mice increased consumption of both chow (706%) and HPD (1279%) pellets during the epochs of light exposure (Extended Data Fig. 2a–d).

In these studies, we tested the effects of VMH^BDNF^ inhibition in animals before and after they were fed a HPD. We noted that at baseline, before optogenetic inhibition, when the DIO mice were later presented with chow, they consumed smaller amounts then they had before they were fed the HPD (Extended Data Fig. 2e). One potential explanation for this finding is that the chow was devalued in the DIO animals fed the HPD^18,19^. However, we found that at baseline before VMH^BDNF^ inhibition, the DIO animals also consumed less of the HPD (Extended Data Fig. 2f). We thus considered that this reduced consumption of the HPD and chow after obesity had developed might have been caused by hyperactive VMH^BDNF^ neurons (as suggested by the increased c-Fos expression). If this hypothesis were true, optogenetic inhibition of VMH^BDNF^ neurons would be expected to have a greater quantitative effect in DIO versus lean animals. Consistent with this, we found that optogenetic inhibition of VMH^BDNF^ neurons using GtACR resulted in a greater quantitative increase in food intake in DIO compared with lean animals (Extended Data Fig. 2g). We interpreted this to mean that VMH^BDNF^ neurons become more active once animals develop diet-induced obesity, at which point they then restrict overeating. (We also imaged these neurons and indeed found increased Ca^2+^ signals in VMH^BDNF^ neurons from DIO mice; see below.) These studies show that VMH^BDNF^ neurons normally restrict intake of both chow and palatable food in DIO animals.

We then assessed whether BDNF itself contributes to these effects by inhibiting BDNF signalling in DIO mice, using a TrkB^F616A^ knock-in mouse carrying a point mutation that renders the receptor sensitive to an allele-specific kinase inhibitor (1-NM PP). Homozygous TrkB^F616A^ mice were fed a HFD, after which they were treated with 1-NM PP1. Mice treated with 1-NM PP1 showed significant increases in body weight (of 17%; Extended Data Fig. 2h) and food intake (of 27%; Extended Data Fig. 2i,j). These data show that BDNF signalling is also required to suppress food intake and weight gain in HPD-fed mice after obesity has developed.

To further explore the mechanism by which VMH^BDNF^ neurons control feeding, we tested whether VMH^BDNF^ neuron activity altered valence, which would suggest a possible role in motivation. However, in an operant conditioning task, VMH^BDNF^ neuron activation failed to entrain a preference for self-inhibition (Extended Data Fig. 2k–m) and also failed to entrain a preference in a flavour conditioning assay (Extended Data Fig. 2n–p). These studies suggest that the effect of VMH^BDNF^ neurons on feeding is not mediated by a general effect on valence. An alternative possibility is that VMH^BDNF^ neurons regulate the consummatory rather than the appetitive phase of feeding. We tested this by analysing whether VMH^BDNF^ neuron inhibition influenced food approach versus food consumption after optogenetic inhibition of VMH^BDNF^ neurons, using two different paradigms: (1) constant photoinhibition for 5 min in an open loop system (Fig. 2i,j); and (2) inhibition in a closed loop system, in which light was delivered only when the head of the mouse was within 3 cm of a chow pellet (Fig. 2k,l). We found that the closed loop configuration led to an approximately three times greater food intake compared with open loop inhibition (Fig. 2l). Consistent with this, the latency of the approach to the pellet was also increased five-fold in the open loop paradigm, showing that VMH^BDNF^ neuron inhibition increased the time taken for mice to reach the food (Fig. 2m). We also noted that open loop VMH^BDNF^ neuron photoinhibition often triggered consummatory behaviour targeted at objects in immediate proximity to the mouse, including the wall and bare floor of the cage. Thus, VMH^BDNF^ neuron photoinhibition not only reduced food approach but seemed to trigger motor behaviours associated with consumption. To characterize this further, we studied the effect of VMH^BDNF^ neuron photoinhibition in the open loop system in the presence of corn cob bedding (Fig. 2n,o). We observed that animals would often chew the bedding during VMH^BDNF^ neuron photoinhibition, and that the presence of bedding further decreased food intake compared with open loop stimulation in a bare cage (Fig. 2p). These data show that VMH^BDNF^ neuron inhibition drives feeding most effectively when food is in proximity to the animal and suggests the possibility that VMH^BDNF^ neurons regulate motor programs required for consumption, independent of caloric value. To further evaluate this, we repeated the closed loop study but provided the mice with a wooden block instead of a chow pellet (Fig. 2q–s). VMH^BDNF^ neuron inhibition in the closed loop led mice to spend around 9% of the trial duration engaged in biting the wooden block (Supplementary Video S1). Taken together, these results indicate that inhibition of VMH^BDNF^ neuron activity may increase food intake by triggering consumption of whatever objects are in proximity (such as wood blocks or corn cob bedding) rather than by altering valence or food-seeking. This finding is analogous to that reported after activation of central amygdala (CeA) projections to the reticular formation, the motor pattern generator for killing bites^20^. Moreover, it raises the possibility that VMH^BDNF^ neurons represent a distal node in the neural circuit regulating feeding, downstream of hedonic and homeostatic drives, that controls the motor components of consumption including biting and chewing. Although we found that a population of BDNF-expressing neurons in the dorsomedial VMH regulates feeding, other studies have identified a separate population of lateral VMH neurons expressing oestrogen receptors and VGlut2 that trigger aggressive biting by males targeted at females^21,22^. We thus tested whether inhibition of VMH^BDNF^ neurons stimulated aggression, using the same assay. However, we did not observe any effect of VMH^BDNF^ neurons on aggressive behaviours (Extended Data Fig. 2q,r). Consistent with the possibility that distinct populations control these behaviours, previous studies have indicated that the VMH^ER^ neurons target the periaqueductal gray as the main output for this bite-based behaviour and have found no convergence with VMH^BDNF^ projections for feeding^23^.

## VMH^BDNF^ neural activity during feeding

**Fig. 3:**
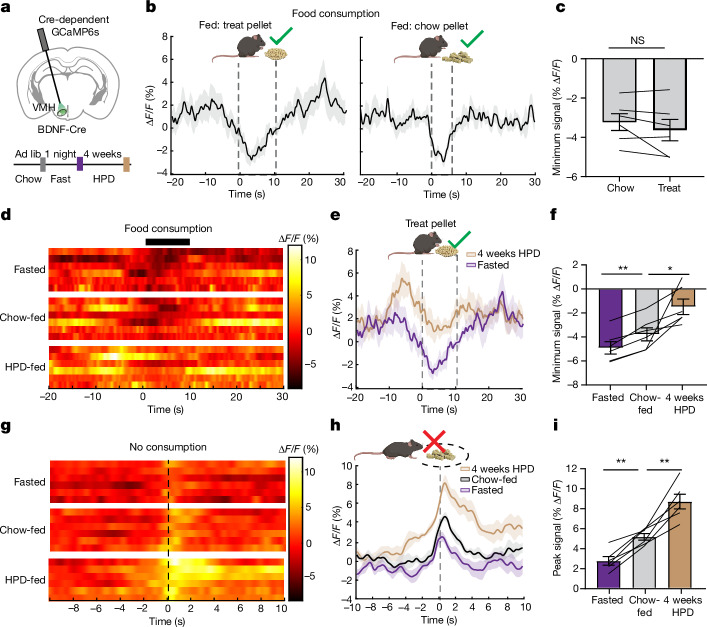
VMH^BDNF^ neuron activity is decreased during food consumption and tuned to energy state. **a**, Schematic of a coronal brain slice with GCaMP expression and fibre placement and (below) recording timeline for energy state manipulations. **b**, Average photometry trace ± s.e.m. (*n* = 6 mice) aligned to a consumption bout of a treat pellet in mice fed chow ad lib (left) or a chow pellet (right). **c**, Comparison of the minimum signal between chow and treat pellet consumption (*n* = 6 mice). **d**, Heatmap of average photometry recordings per mouse (*n* = 6 mice) aligned to a bout of treat pellet consumption. **e**, Average overall photometry trace ± s.e.m. (*n* = 6 mice) aligned to a bout of treat pellet consumption in fasted (purple) and 4 weeks HPD-fed (beige) mice. **f**, Comparison of the minimum signal during treat consumption in *n* = 6 mice. **g**, Heatmap of average photometry recordings per mouse (*n* = 6 mice) aligned to food approach without consumption. **h**, Overall average overall photometry (*n* = 6 mice) aligned food approach without consumption in fasted (purple) and 4 weeks HPD-fed (beige) mice. **i**, Comparison of the maximum signal. Paired *t*-test was used in **c**, and a mixed-effects model (restricted maximum likelihood) with Holm–Šídák’s multiple comparisons test was used in **f** and **i**. *P < 0.05, **P < 0.01, ***P < 0.001, ****P < 0.0001. Error bars indicate s.e.m. Elements in **b**, **e** and **h** were created using BioRender (https://biorender.com). **a** is adapted from ref. ^56^, Elsevier. Photographs of food pellets in **b**,**e**,**h** are from Bio-Serv. Source Data

The potent effect by which VMH^BDNF^ neurons reduced feeding suggested that the activity of these neurons might be decreased as the animals consumed food and thus become permissive for feeding. We measured the in vivo activity of VMH^BDNF^ neurons using fibre photometry to monitor intracellular calcium levels when animals consumed or rejected food. BDNF–Cre mice received injections of a Cre-dependent AAV encoding GCamp6s into the VMH (Fig. 3a), and calcium signals were measured during bouts of feeding. Time-locked recordings showed a significant (4%) decrease in VMH^BDNF^ neuron activity coinciding with consumption of a chow pellet (Fig. 3b,c and Supplementary Video S2). This was consistent with the optogenetic results and suggests that VMH^BDNF^ neuron activity gates feeding behaviour and needs to be inhibited for consumption to commence. To test whether the activity of VMH^BDNF^ neurons was influenced by food palatability, we recorded their response during consumption of sucrose treat pellets; this revealed a decrease of similar magnitude to that seen after chow consumption (Fig. 3b,c).

**Extended Data Fig. 3:**
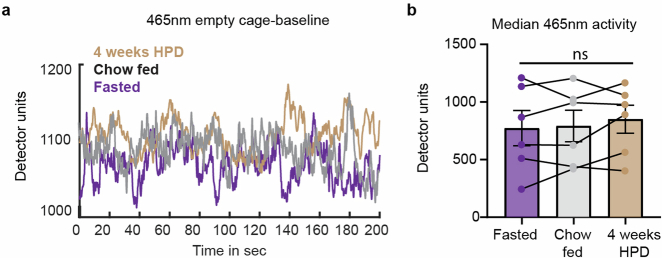
Detector unit counts of photometry mice in fed, fasted and DIO state. **a**, 200 s long example traces in detector units (not normalized) during empty cage exploration and **b**, comparison of the median thereof. Repeated measures one-way ANOVA. *P < 0.05, **P < 0.01, ***P < 0.001, ****P < 0.0001. Error bars ± s.e.m. Source Data

Having established that VMH^BDNF^ neuron activity was reduced during the consumption of chow and sucrose pellets, we investigated the effects of alterations in energy balance. Neural activity was analysed in three sets of conditions: after an overnight fast and in mice fed a chow or a HPD for 4 weeks (Fig. 3a). The baseline activity of these neurons in an empty home cage showed no difference among these three conditions (Extended Data Fig. 3a,b). However, recordings of the activity of VMH^BDNF^ neurons during bouts of feeding revealed an approximately 60% higher level of activity of VMH^BDNF^ neurons in the DIO mice versus chow-fed animals (Fig. 3d–f). In addition, the activity in VMH^BDNF^ neurons was around 30% lower in fasted versus chow-fed animals. We also observed that when chow-fed animals approached a chow pellet within a radius of 2 cm but did not consume it, the calcium signal in VMH^BDNF^ neurons significantly increased (Fig. 3g–i and Supplementary Video S3). There was also an increase in VMH^BDNF^ activity when fasted mice approached but did not consume the food. However, in this case, the increased activity was only half that observed when fed mice approached but did not consume the pellet, and only 30% of that when DIO mice did not consume it (Fig. 3i). In aggregate, these data show that VMH^BDNF^ neural activity is inversely correlated with food consumption, and that activity responses are tuned to the energy state of an animal.

## VMH^BDNF^ neurons are downstream of the Arc

**Fig. 4:**
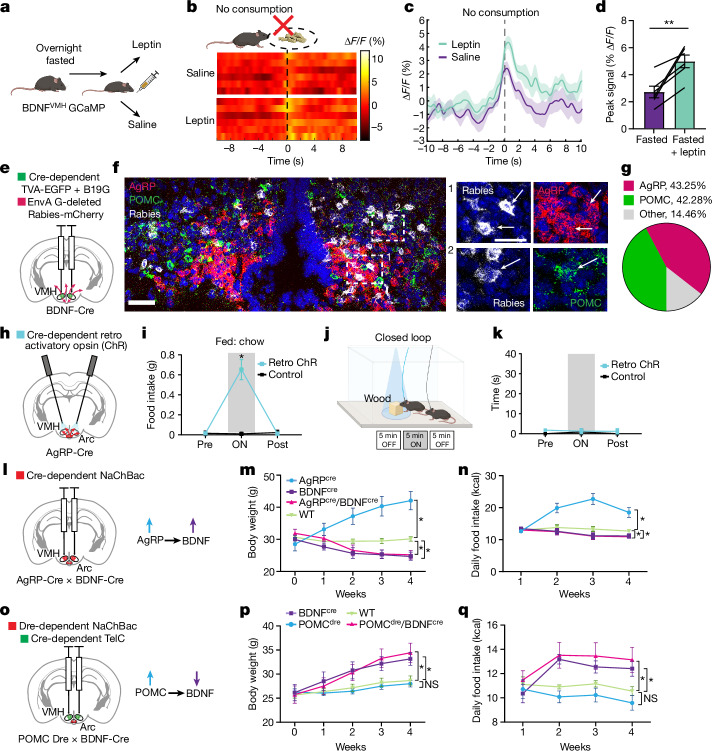
VMH^BDNF^ neurons are anatomically and functionally downstream of AgRP and POMC neurons. **a**, Schematic of experimental design. **b**,**c**, Heatmap of average photometry recordings of six mice (**b**) and overall average photometry trace (±s.e.m.) (*n* = 6 mice) aligned to food approach without consumption after injection with saline (purple) or leptin (green) (**c**). **d**, Comparison of the peak photometry signal. **e**, Schematic of retrograde viral tracing approach. **f**, Representative example (*n* = 3 mice) of a coronal Arc section with in situ hybridization for Rabies (white), AgRP (red) and POMC (green) and magnification examples of boxed areas 1 and 2. **g**, Quantification of average overlap of Rabies-labelled cells with AgRP and POMC in the Arc (*n* = 3 mice). **h**, Schematic of retrograde ChR expression in Arc and fibre placement in VMH. **i**, Food intake of ad lib fed ChR (*n* = 4) and control mice (*n* = 4) tested with chow. **j**, Schematic of closed loop optogenetic inhibition with a wood block and timeline. **k**, Quantification of percentage of time spent biting the wood block for ChR (*n* = 4) and control mice (*n* = 4). **l**, Schematic of bilateral expression of Cre-dependent NaChBac in Arc and change in neural activity. **m**,**n**, Weekly time course of body weight (**m**) and calorie intake (**n**) of AgRP–Cre (*n* = 6 mice), BDNF–Cre (*n* = 6 mice), AgRP/BDNF–Cre mice (*n* = 6 mice) and control mice (*n* = 7 mice) after NaChBac injection. **o**, Schematic of bilateral expression of Dre-dependent NaChBac in Arc and Cre-dependent TelC in VMH and respective changes in neuronal activity. **p**,**q**, Weekly time course of body weight (**p**) and calorie intake (**q**) of POMC–Dre (*n* = 6 mice), BDNF–Cre (*n* = 7 mice), POMC–Dre/BDNF–Cre (*n* = 6 mice) and control (*n* = 8 mice) mice after AAV injection. Paired *t*-test was used in **d**, and two-way RM ANOVA with Šídák’s multiple comparisons test was used in **i**, **k**, **m**, **n**, **p** and **q**. *P < 0.05, **P < 0.01, ***P < 0.001, ****P < 0.0001. Error bars indicate s.e.m. Scale bars, 50 µm (**f**, left), 30 µm (**f**, right). Elements in **a**, **b** and **j** were created using BioRender (https://biorender.com). **e**, **h**, **l** and **o** are adapted from ref. ^56^, Elsevier. Photograph of food pellets in **b** is from Bio-Serv. Source Data

**Extended Data Fig. 4:**
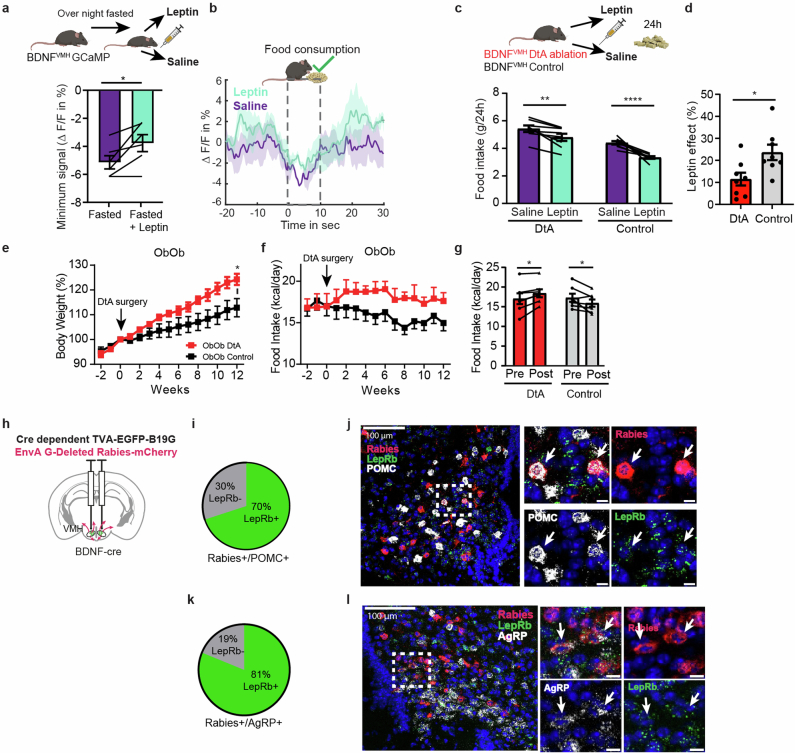
Leptin engages VMH^BDNF^ neurons to suppress feeding. **a**, Schematic of experimental design, below, comparison of the average photometry signal during food consumption between 6 mice treated with leptin and saline (paired t-test). **b**, Average photometry trace ( ± sem) of mice injected with saline (purple) or leptin (green) (n = 6 mice) aligned to a bout of treat pellet consumption. **c**, Schematic of experimental design and below food intake in 24 h after a leptin or saline ip injection in Dta and control mice (n = 8 each group) (DtA: p = 0.0031, Ctrl: p < 0.0001, two-way ANOVA with Holm-Šídák’s multiple comparisons test). **d**, Quantification of the feeding suppressive effect of leptin by normalizing to the saline injected food intake (p = 0.0187, Welch’s t-test). **e**, Weekly time course of body weight (at week 12: p = 0.0248, Welch’s t-test) and **f**, calorie intake of chow fed ob/ob mice crossed to BDNF cre (n = 7) and ob/ob control mice (n = 7) injected with DtA with quantification of average food intake in **g** before and after DtA ablation (P = 0.036, two-way RM ANOVA with Šídák’s multiple comparisons test). **h**, Schematic of G-deleted pseudotyped rabies and helper AAV expression. **i**, Quantification of average co-expression of rabies labelled POMC neurons with LepRb of n = 3 mice. **j**, Representative example (n = 3 mice) of a coronal Arc section with in situ hybridization for POMC (white), rabies (red) and LepRb (green), right, high magnification examples of dotted square with arrows indicating triple labelled neurons, scale bar = 10 µm. **k**, Quantification of average co-expression of rabies labelled AgRP neurons with LepRb of n = 3 mice. **l**, Representative example of a coronal Arc section with in situ hybridization for AgRP (white), rabies (red) and LepRb (green); right, high magnification examples of dotted square with arrows indicating triple labelled neurons, scale bar = 10 µm. *P < 0.05, **P < 0.01, ***P < 0.001, ****P < 0.0001. Error bars ± s.e.m. Source Data

Our finding that VMH^BDNF^ neural dynamics were altered by energy state—that is, in fed, fasted and DIO animals—raised the possibility that they might respond to adipocyte-derived hormone leptin^24,25^. This was tested using fibre photometry recordings after leptin versus saline injections into fasted animals (Fig. 4a–d). Leptin increased VMH^BDNF^ neural activity by around 80% when animals approached but did not consume a chow pellet (Fig. 4d) and also increased it by around 25% when animals consumed a treat pellet in comparison with saline-injected mice (Extended Data Fig. 4a,b). To confirm that these neurons contributed to the effect of leptin on food intake, we treated animals with leptin (3 mg kg^−1^, intraperitoneally) after VMH^BDNF^ neural ablation with DtA (Extended Data Fig. 4c,d). The reduction in food intake caused by leptin was significantly decreased in animals in which VMH^BDNF^ neurons were ablated. To test whether VMH^BDNF^ neurons could respond to signals besides leptin, we crossed BDNF–Cre mice to leptin-deficient *ob*/*ob* mice and injected DtA into the VMH (Extended Data Fig. 3e–g). The *ob*/*ob* mice with VMH^BDNF^ ablation showed significantly increased food intake and became more obese. As *ob*/*ob* mice lack leptin, these data indicate that VMH^BDNF^ neurons sense satiety signals in addition to leptin.

**Extended Data Fig. 5:**
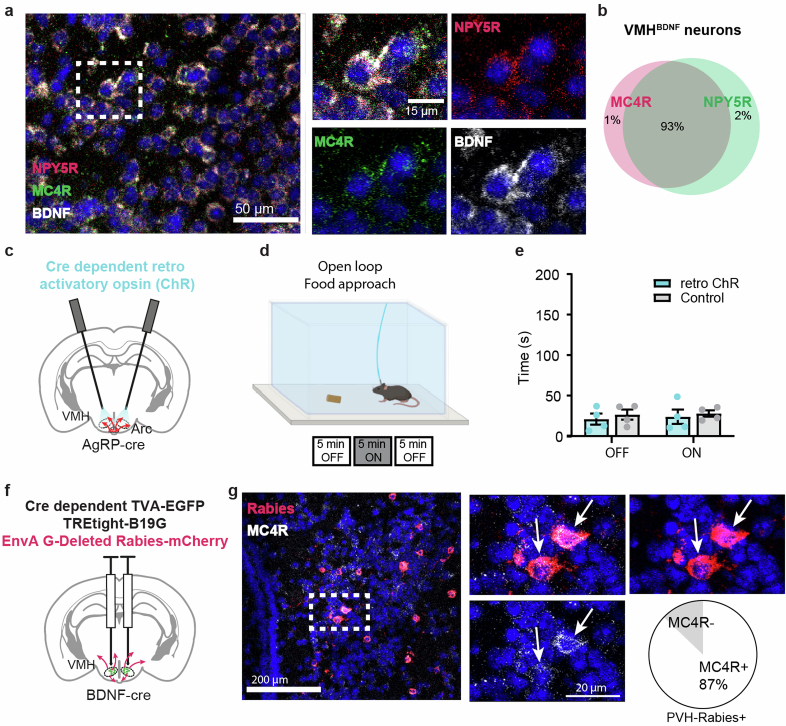
VMH^BDNF^ neurons are downstream of melanocortin signalling. **a**, Representative example (n = 3 mice) of a coronal VMH section with in situ hybridization for BDNF (white), MC4R (red) and NPY5R (green), right, high magnification examples of overlap. **b**, Quantification of average co-expression of VMH^BDNF^ neurons (of n=3 mice) with MC4R and NPY5R. **c**, Schematic of retrograde ChR expression in Arc and optic fibre placement in VMH and **d**, schematic of open loop optostimulation for food approach assessment. **e**, Quantification of latency to reach a food pellet of AgRP cre mice injected with retro ChR into the VMH (n = 4) and control mice (n = 4). Two-way RM ANOVA with Šídák’s multiple comparisons test. **f**, Schematic of G-deleted pseudotyped rabies and helper AAV injection. **g**, Representative example of a coronal Arc section with in situ hybridization for MC4R (white) and rabies (red); right, high magnification examples of overlap and quantification of n = 3 mice. *P < 0.05, **P < 0.01, ***P < 0.001, ****P < 0.0001. Error bars ± s.e.m. Source Data

Previous anatomic^26^ and single-cell RNA sequencing studies^16^ have indicated that VMH^BDNF^ neurons do not express leptin receptors, suggesting that the effects of leptin on these neurons are indirect. Therefore, we next determined whether VMH^BDNF^ neurons received inputs from the Arc (Fig. 4e), a key site of action for multiple interoceptive signals including leptin. The inputs to VMH^BDNF^ neurons were labelled by monosynaptic tracing with pseudotyped Rabies. We injected helper AAVs and G-deleted Rabies-mCherry viruses into the VMH of BDNF–Cre mice and observed prominent inputs from the Arc, where leptin-responsive proopiomelanocortin (POMC) and AgRP neurons are located (Fig. 4f,g). We next performed multichannel fluorescence in situ hybridization (FISH) using probes for Rabies, POMC and AgRP; this revealed that a large proportion of Arc inputs were from either POMC (42.28 ± 7.95%) or AgRP (43.25 ± 3.53%) neurons. In addition, FISH with a LepR probe revealed that many of the Rabies-labelled AgRP and POMC neurons also expressed the leptin receptor (Extended Data Fig. 4h–l). Consistent with previous studies^27,28^, in situ hybridization also showed that 93 ± 2.29% VMH^BDNF^ neurons coexpressed both neuropeptide Y receptor 5 (NPY5R) and melanocortin receptor 4 (MC4R), suggesting that VMH^BDNF^ neurons are a point of convergence for Arc^POMC^ and Arc^AgRP^ outputs (Extended Data Fig. 5a,b).

We next investigated whether the AgRP projections to the VMH could drive feeding, similar to the effect of somatic activation of AgRP neurons in the Arc^29^. A Cre-dependent retrograde AAV encoding ChR was injected into the VMH of AgRP–Cre mice, and optogenetic fibres were implanted above the VMH (Fig. 4h). AgRP neurons are GABAergic, and optogenetic activation of the AgRP projections to the VMH increased food intake in chow-fed mice (Fig. 4i) but did not cause any decrease in food approach (Extended Data Fig. 5c–e). Moreover, closed loop activation targeted at a wood block did not elicit motor sequences of consummatory behaviour (Fig. 4j,k), suggesting that although projection-specific activation regulates food intake, activation of these inhibitory inputs by themselves is not of sufficient intensity to activate motor programs of food consumption. This is in contrast to the effect observed when inhibiting the soma of VMH^BDNF^ neurons. AgRP projections to the paraventricular hypothalamus (PVH) have also been reported to increase food intake through MC4R-expressing neurons^30,31^, and retrograde tracing from VMH^BDNF^ neurons also showed inputs from the PVH. In addition, in situ hybridization of PVH neurons for MC4R after retrograde tracing revealed that VMH^BDNF^ neurons also receive monosynaptic inputs from PVH^MC4R^ neurons (Extended Data Fig. 5f,g). Thus, melanocortin pathways involving neurons from multiple sites converge on VMH^BDNF^ neurons.

To assess whether VMH^BDNF^ neurons were functionally downstream of interoceptive neurons in the Arc, we simultaneously activated both Arc^AgRP^ and VMH^BDNF^ neurons by expressing NaChBac in one or both populations (Fig. 4l–n). NaChBac is a bacterial sodium channel that leads to constitutive neural activation. AgRP–Cre mice were crossed to BDNF–Cre mice, and an AAV expressing a floxed NaChBac was stereotactically injected into the Arc and VMH (Fig. 4l). Consistent with previous results^32^, activation of Arc^AgRP^ neurons alone led to marked hyperphagia and obesity. Constitutive activation of VMH^BDNF^ neurons alone significantly reduced food intake and body weight. However, simultaneous activation of both populations in double-positive AgRP–Cre/BDNF–Cre mice fully recapitulated the effect seen with BDNF activation alone, with these animals showing significantly decreased food intake and weight. These data show that VMH^BDNF^ neurons are functionally downstream of Arc^AgRP^ neurons and thus represent an important output site.

The functional relationship between Arc^POMC^ and VMH^BDNF^ neurons was tested by crossing POMC–Dre mice to BDNF–Cre mice and injecting a Dre-dependent NaChBac into the Arc and a Cre-dependent TelC virus into the VMH (Fig. 4o). TelC prevents vesicular release and thus silences neurons. Consistent with the effect of DtA ablation of VMH^BDNF^ neurons, BDNF–Cre mice expressing TelC alone showed significantly increased food intake and body weight (Fig. 4p,q). Constitutive activation of Arc^POMC^ neurons led to slight decreases in food intake and body weight, consistent with previous studies^33^. Here, again, the phenotype of the POMC–Dre/BDNF–Cre double-positive mice recapitulated the phenotype of mice with constitutive inhibition of VMH^BDNF^ neurons alone. These data show that VMH^BDNF^ neurons are also functionally downstream of Arc^POMC^ neurons. Overall, this set of experiments showed that VMH^BDNF^ neurons receive inputs from interoceptive neurons in the Arc and are functionally downstream of the melanocortin system, as previously suggested^28^. We next determined where they projected to and further assessed their effects on consummatory behaviours.

## VMH^BDNF^ neurons project to brainstem

**Fig. 5:**
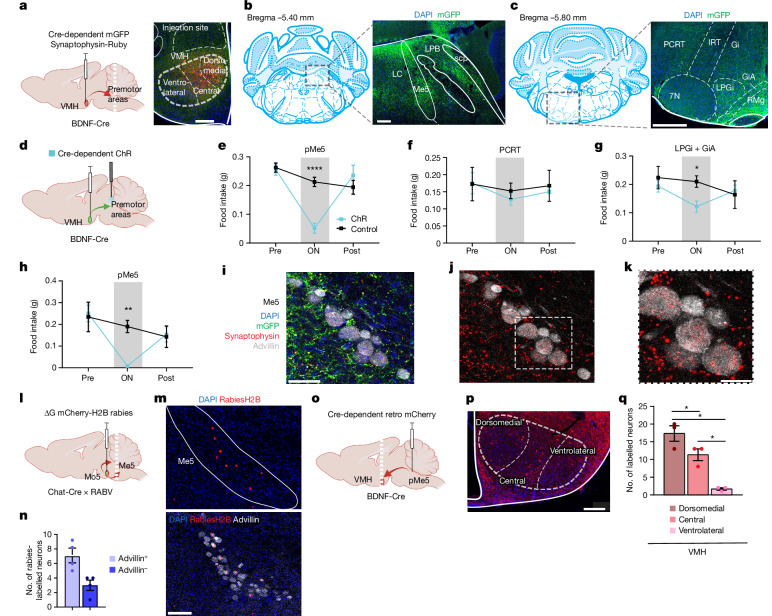
Activation of VMH^BDNF^ neuron projections to premotor areas. **a**, Left, schematic of a sagittal section with mGFP–synaptophysin–Ruby expression. Right, representative image (*n* = 3 mice) of injection site. **b**, Brain atlas with square indicating the section on the right: representative (*n* = 3 mice) coronal section of mGFP expression and DAPI (blue) of Me5. scp, superior cerebellar peduncles. **c**, Brain atlas with dotted square indicating the imaged section on the right: representative (*n* = 3 mice) coronal section of mGFP expression and DAPI (blue). IRT, intermediate reticular nucleus; 7N, cranial nerve 7; RMg, raphe magnus nucleus. **d**, Schematic of a sagittal section with ChR expression. **e**–**g**, Chow intake of overnight fasted mice with implants above pMe5 (*n* = 7 mice each group; **e**), PCRT (*n* = 4 mice per group; **f**), or LPGi + GiA (*n* = 5 mice each group; **g**). **h**, Ad lib fed mice tested with HPD ChR (*n* = 7) and control mice (*n* = 7). **i**, Representative (*n* = 3 mice) coronal image of Me5 after mGFP–synaptophysin–Ruby injection in VMH (see **a**) with projections (green), synaptophysin (red) and immunofluorescence staining for advillin (white). **j**, Same image as in **i** but with advillin (white) and synaptophysin (red) only. **k**, Enlargement of the square in **j**. **l**, Schematic of a sagittal section with retrograde tracer injection into Mo5. **m**, Representative coronal image (*n* = 4 mice) of Me5 with Rabies-labelled nuclei (red) and overlay with advillin immunofluorescent staining (white) (below). **n**, Quantification of Rabies-labelled neurons (*n* = 4 mice) colabelled with advillin. **o**, Schematic of a sagittal section with retro-tracer injection into pMe5. **p**, Representative image (*n* = 3 mice) of VMH with mCherry-labelled BDNF neurons (red) and DAPI. **q**, Quantification of mCherry-labelled neurons in the dorsomedial, central and ventrolateral VMH. Two-way RM ANOVA with Šídák’s multiple comparisons test was used in **e**–**h**. Mixed-effects analysis with Holm–Šídák’s multiple comparisons test was used in **q**. *P < 0.05, **P < 0.01, ***P < 0.001, ****P < 0.0001. Error bars indicate s.e.m. Scale bars, 200 µm (**a**), 100 µm (**b**,**m**), 400 µm (**c**), 50 µm (**i**), 20 µm (**j**), 200 µm (**p**). Elements in **a**, **d**, **l** and **o** were created using BioRender (https://biorender.com). **b** and **c** are adapted from ref. ^56^, Elsevier. Source Data

**Extended Data Fig. 6:**
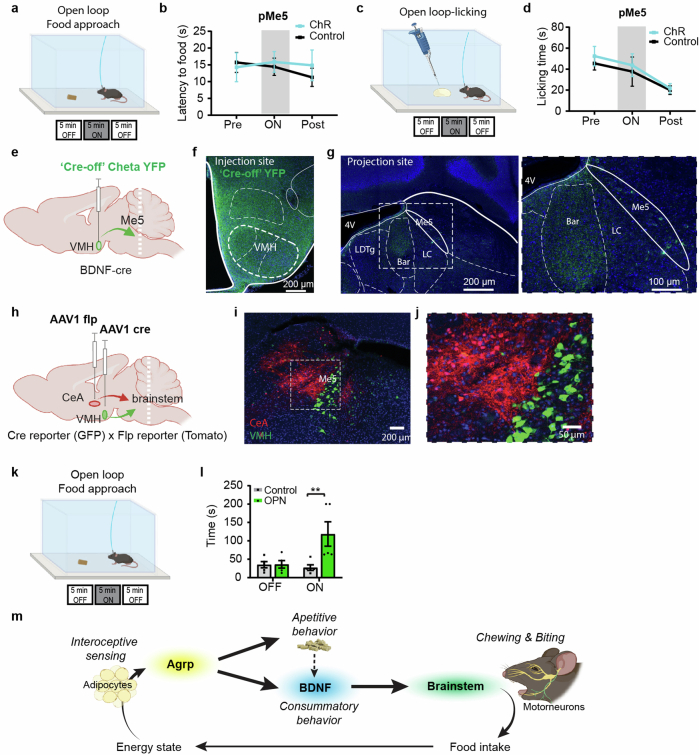
VMH^BDNF^ neuron projections to brainstem drive solid food intake. **a**, Schematic of open loop optogenetic activation with ChR and experimental timeline. **b**, Latency to approach the chow pellet in ChR and Ctrl mice (n = 7 mice each group). Two-way RM ANOVA with Šídák’s multiple comparisons test. **c**, Schematic of open loop optogentic activation and experimental timeline for licking measurements. **d**, Quantification of time spent licking of ChR (n = 7) and control mice (n = 6) given 50 µl liquid diet in an open loop inhibition setup. ChR: 43.71 ± 10.84 s, two-way RM ANOVA with Šídák’s multiple comparisons test. **e**, Schematic of a ‘cre out’ Cheta YFP virus injection into VMH of BDNF cre mice, labelling non-BDNF neurons. **f**, Representative image (n = 4 mice) of a coronal section of the injection site and **g**, image of projections to Barrington nucleus and surrounding areas and high magnification of area in dotted square. Me5 was marked by autofluorescence of neurons in this nucleus. 4 V = fourth ventricle, Bar = Barrington’s nucleus, LDTg = Laterodorsal tegmental nucleus. **h**, Schematic of virus injection into the CeA and VMH of Cre and Flp reporter expressing mice. **i**, Representative example of n = 3 mice of brainstem premotor areas (mesencephalic) and **j**, high magnification of AAV1 labelled neurons. **k**, Schematic of open loop optostimulation for food approach assessment. **l**, Quantification of latency to reach a food pellet of BDNF cre mice injected with eOPN3 into VMH and fibres implanted above pMe5 (n = 5) and control mice (n = 5). Two-way RM ANOVA with Šídák’s multiple comparisons test. **m**, Schematic of a proposed circuit for energy state driven food consumption behaviour. *P < 0.05, **P < 0.01, ***P < 0.001, ****P < 0.0001. Error bars ± s.e.m. Source Data

We mapped the projection sites of VMH^BDNF^ neurons by injecting an AAV with a Cre-dependent mGFP–synaptophysin–Ruby gene into the VMH of BDNF–Cre mice (Fig. 5a–c). Fluorescence imaging of neurons expressing mGFP revealed dense projections to several known premotor sites^20,34–37^ in the brainstem that have previously been shown to send projections on to motor neurons of the jaw muscle and tongue; these included the mesencephalic nucleus (Me5), lateral paragigantocellular (LPGi) and gigantocellular reticular nucleus alpha part (GiA), with only weak projections to the parvocellular reticular formation (PCRT). We next assessed the function of each of these VMH^BDNF^ neuron-to-premotor projections by optogenetically activating VMH^BDNF^ nerve terminals there. Optical fibres were implanted above the peri-Me5 (pMe5), LPGi and GiA, and PCRT in animals expressing ChR in VMH^BDNF^ neurons (Fig. 5d–g), and feeding of a chow pellet was tested after an overnight fast. Projections to the LPGi and GiA were of interest, as the LPGi–motor connection develops during weaning and might therefore be involved in solid food consumption. However, activation had only a modest effect on feeding, reducing it by 37%, whereas projections to the PCRT, which has been reported to stimulate the killing bite in crickets, failed to suppress feeding at all. By contrast, projections to pMe5 significantly reduced feeding (by 80%), similar to the effect we observed when stimulating VMH^BDNF^ soma. In addition, activation of pMe5 projections suppressed consumption of HPD feeding (Fig. 5h) and was specific to solid foods, as it did not affect liquid diet licking (Extended Data Fig. 6c,d). Moreover, pMe5 projections specifically control food consumption and not approach, as inhibition of the terminals did not shorten the latency of the approach to a chow pellet (Extended Data Fig. 6a,b).

We next characterized the inputs and outputs from Me5 more fully. Me5 is a small nucleus; to further confirm that VMH^BDNF^ neurons project there, we performed immunofluorescence for advillin, a marker of Me5 neurons^38^, together with Ruby-tagged synaptophysin introduced into VMH^BDNF^ neurons as above (Fig. 5i–k). Dense synapses were found in Me5 and on advillin-expressing cells, as well as cells in Me5 that did not express advillin. Monosynaptic retrograde Rabies tracing from Chat–Cre-expressing neurons in the trigeminal motor nucleus (Mo5) confirmed that in Me5 both advillin and non-advillin neurons project to motor neurons in Mo5 (Fig. 5l–n).

Next, we performed retrograde tracing from pMe5 by injecting a retro-AAV expressing a Cre-dependent mCherry into the pMe5 of BDNF–Cre mice. In line with the c-Fos data (Fig. 1a), we found that most BDNF neurons projecting to pMe5 were located in the ventromedial and central parts of the VMH project (Fig. 5o–q). We also investigated whether the other non-BDNF VMH populations projected to pMe5 using a ‘Cre-off’ strategy (Extended Data Fig. 6e–g). In this experiment, we used an AAV in which Cre turns off a eYFP cassette such that it will be constitutively expressed in neurons that do not express Cre. A ‘Cre-off’ AAV encoding eYFP-ChR was injected into the VMH of BDNF–Cre mice, and the projection sites were ascertained. The non-BDNF neurons in VMH showed only minimal projections in the Barrington nucleus and locus coeruleus and failed to show projections to pMe5.

To investigate potential inputs to pMe5 from extrahypothalamic areas with known premotor effects on jaw movements, we tested for projections from the CeA. CeA neurons control the killing bite of crickets during prey-hunting and have been reported to project to the PCRT^20^. However, we did not find any further inputs to pMe5 from the CeA, using two anterograde tracing AAV1 viruses encoding either Flp or Cre. In this study, the Flp-expressing AAV1 was injected into the CeA and the Cre AAV1 was injected into the VMH of mice carrying both a Cre-dependent GFP and Flp-dependent tdTomato reporter (Extended Data Fig. 6h–j). With this approach, CeA projections appear red and VMH projection neurons appear green. As before, the VMH projection neurons were localized primarily in pMe5, whereas the CeA projections were seen in PCRT neurons, consistent with a previous report^20^, and in the medial and lateral parabrachial nucleus next to pMe5 (Extended Data Fig. 6i,j). Our finding that the targets of the VMH^BDNF^ neurons are different from CeA is consistent with the finding^20^ that the CeA → PCRT circuit does not alter food consumption.

## VMH^BDNF^ → pMe5 regulates jaw movements

**Fig. 6:**
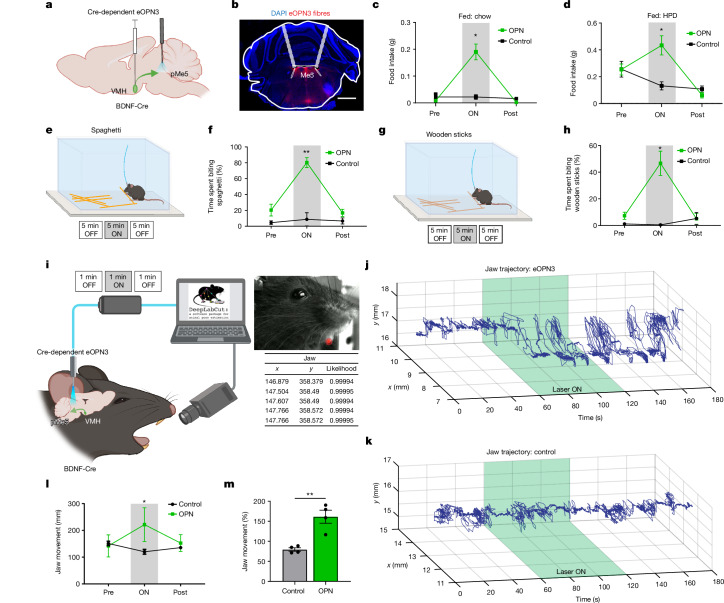
Inhibition of VMH^BDNF^ projections to Me5. **a**, Schematic of a sagittal brain with eOPN3 expression in the VMH and fibre placement above pMe5. **b**, Representative image (*n* = 5 mice) of eOPN3–Ruby expression and fibre placement above Me5. **c**,**d**, Food intake of ad lib fed mice tested with chow (**c**), and ad lib fed eOPN3 (*n* = 5) and control mice (*n* = 5) tested with HPD (**d**). **e**, Schematic of constant optogenetic inhibition during spaghetti feeding and experimental timeline. **f**, Quantification of time spent biting spaghetti of ad lib fed mice (*n* = 4). **g**, Schematic of constant optogenetic inhibition during wood-stick biting and experimental timeline. **h**, Quantification of time spent biting wood sticks of ad lib fed mice (*n* = 4). **i**, Schematic of high-speed jaw-tracking in head-fixed mice with concurrent optogenetic inhibition and subsequent pose estimation. **j**, Representative example (*n* = 4 mice) of jaw movements in two dimensions over time of an eOPN3-expressing mouse with the laser-on time indicated in green (60–120 s). **k**, Representative example (*n* = 4 mice) of jaw movements in two dimensions over time of a control mouse with the time of laser stimulation indicated in green (60–120 s). **l**, Quantification of absolute jaw movements during laser on and off periods in four control and four eOPN3 mice. **m**, Comparison of jaw movement between control and eOPN3 mice after normalization to laser-off period. Two-way RM ANOVA with Šídák’s multiple comparisons test in **c**, **d**, **f**, **h**, **l**. Unpaired *t*-test in **m**. *P < 0.05, **P < 0.01, ***P < 0.001, ****P < 0.0001. Error bars indicate s.e.m. Scale bar, 1,000 µm. Elements in **a**, **e**, **g** and **i** were created using BioRender (https://biorender.com). Source Data

We next investigated whether inhibition of the VMH^BDNF^ projections to pMe5 recapitulated the effects seen during inhibition of VMH^BDNF^ soma (Fig. 2e–h). A Cre-dependent AAV encoding eOPN3, an inhibitory mosquito-derived rhodopsin, was injected into the VMH of BDNF–Cre mice, and implants were placed above the pMe5 projection sites (Fig. 6a,b). Inhibition of the VMH^BDNF^ projections to pMe5 increased food intake of both chow (863%) and HPD (170%), with a similar magnitude to that seen after inhibition of the soma (Fig. 6c,d). Of note, eOPN3 is a GPCR and thus does not elicit the instantaneous inhibition seen after photoinhibition using GtACR, precluding studies of inhibition in a closed loop paradigm. However, constant inhibition (light) in an open loop configuration decreased food approach (329% latency; Extended Data Fig. 6k,l) and increased chewing of spaghetti and non-nutritious wooden sticks (wood: 642%, spaghetti: 394%; Fig. 6e–h and Supplementary Videos 4–6). This suggested that inhibition of VMH^BDNF^ projections to pMe5 might directly regulate consummatory actions, including movement of the jaw.

We directly assessed jaw movements in the absence of food or other stimuli in head-fixed mice using a high-speed side camera during optogenetic inhibition of the VMH^BDNF^ projections to Me5. Pose estimation of the jaw position was performed with DeepLabCut (Fig. 6i). Photoinhibition of VMH^BDNF^ terminals in pMe5 using eOPN3 reproducibly evoked rhythmic jaw movements, with a similar time course to that reported in studies of food intake (Fig. 6j,k and Supplementary Video 7). By contrast, control mice without eOPN3 expression showed infrequent jaw movements (Supplementary Video 8). Photoinhibition of VMH^BDNF^ terminals in pMe5 led to an increase of approximately 160% in jaw movements during laser inhibition compared with controls (Fig. 6l,m).

## Discussion

A key function of the brain is to generate adaptive behaviours in response to an array of interoceptive and sensory inputs. A full understanding of how behaviours are controlled in higher organisms thus requires the elucidation of neural circuits linking these inputs to motor outputs. In this work, we show that BDNF neurons in the VMH convey inputs from an interoceptive node to motor outputs associated with feeding. These neurons receive direct monosynaptic inputs from AgRP and POMC neurons in the Arc, two key interoceptive populations. In vivo imaging studies show that the activity of VMH^BDNF^ neurons is inversely correlated with feeding, tuned to energy state and modulated by leptin. Optogenetic activation of VMH^BDNF^ neurons reduces feeding, whereas inhibiting them increases food intake. In the absence of food, these actions are directed at inedible objects such as wood blocks or sticks, suggesting that they drive motor sequences associated with feeding. Inhibition of VMH^BDNF^ projections to pMe5, a known premotor site, elicits rhythmic activation of the jaw muscles even in the absence of food. In aggregate, these data show that VMH^BDNF^ neurons directly connect neurons receiving interoceptive inputs in the Arc to a premotor site that controls jaw movements, thus defining a simple subcortical circuit that regulates food consumption (Extended Data Fig. 6m).

Complex behaviours such as feeding, which are context dependent and thus variable, are considered to be mechanistically distinct from reflexes in which a defined stimulus generally results in an invariant response^4^. However, as suggested by Sherrington in 1906^1^, the ‘transition from reflex action to volitional control is not abrupt and sharp’. Sherrington pointed out that reflexes are also under volitional control, noting that the cough reflex (and others) can be ‘checked, released, or modified in its reaction with such variety and seeming independence of external stimuli by the existence of a spontaneous internal process expressed as will’. The identification of an Arc → VMH^BDNF^ → pMe5 circuit reported here is consistent with Sherrington’s hypothesis. Moreover, this line of reasoning suggests the possibility that even among mammals, feeding and other complex behaviours might be controlled in part by simple, reflex-like circuits that are in turn modulated by descending inputs from higher centres, as has also been suggested by Crick and Koch^39^. By identifying pMe5 as a premotor node regulating consummatory behaviours, this circuit potentially provides a framework for establishing a hierarchy among the many previously identified nodes that have been shown to control food intake^40^. Although the identification of this circuit does not preclude the possibility that other premotor sites may also regulate feeding, our finding that VMH^BDNF^ projections to pMe5 regulate consummatory behaviours is consistent with studies of mice with Me5 lesions as a result of Drg11 knockout. The Drg11-knockout mice starved to death at weaning owing to an inability to consume solid food but survived if provided with liquid nutrient^41^. Our finding of a premotor site regulating consummatory behaviours thus raises the possibility that other innate behaviours are controlled by premotor sites elsewhere. Tinbergen suggested that key nodes regulating motor patterns are released from inhibition in response to sensory and interoceptive inputs^42^, and our identification of a node regulating food consumption may therefore have general implications for how specific behaviours are selected to the exclusion of other competing behaviours^2^.

The data reported here also show that VMH^BDNF^ neurons are downstream of AgRP neurons, which play an essential part in driving food intake when an animal’s energy state is low. AgRP levels are suppressed by leptin^43^, and our findings thus suggest that the obesity seen with mutations of the genes encoding BDNF, TrkB, leptin, the LepR receptor and melanocortin is caused by altered function of the same expanded feeding circuit. Whereas AgRP neuron activation by itself normally drives feeding, concurrent VMH^BDNF^ neuron activation entirely blocks this. AgRP neurons act as interoceptive sensors of changes in energy state and drive all aspects of hunger^29,44^, including both appetitive behaviours such as food-seeking and consummatory behaviours. However, we found that AgRP inputs to VMH^BDNF^ neurons only increased the consummatory phase of feeding and did not alter food approach, suggesting that different projection sites of these neurons regulate the motivational component. This is consistent with a hierarchical architecture for innate behaviours, as proposed by Craig^45^ and Lorenz^3^—and especially Tinbergen^2^, who suggested that appetitive and consummatory behaviours are controlled by different sites, and that the initiation of the consummatory phase only begins when the appetitive phase is completed and food is in proximity. Moreover, AgRP neuron projections to the VMH did not drive the consummatory motor sequences by themselves in the absence of food, suggesting that further signals are necessary to ‘release’ the motor programs necessary for mastication. As suggested by Tinbergen, such inhibition could result from sensory information derived from potential food sources, signalling that food is in proximity. The possibility that AgRP inputs to VMH^BDNF^ neurons are not sufficient to activate consumption is analogous to Tinbergen’s experiment in which honey bees only landed on colourful artificial flowers when these were paired with appropriate odours^42^.

The data also show that VMH^BDNF^ neurons represent a critical node downstream of leptin signalling, as demonstrated by our findings that leptin increases the gain of these neurons, according to fibre photometry, and that ablating VMH^BDNF^ neurons increases the weight of leptin-deficient *ob*/*ob* mice. Retrograde viral tracing from VMH^BDNF^ neurons identified inputs from AgRP and POMC in the Arc, the site of a circumventricular organ; these convey the leptin signal to VMH^BDNF^ neurons, which themselves do not express leptin receptors. Constitutive alterations in VMH^BDNF^ neural activity entirely blocked the effects of both POMC or AgRP neural activation on feeding, confirming that they are key functional target of these neurons. This finding is in line with those of prior studies reporting the presence of POMC and AgRP fibres and terminals in the VMH^28,46^ and with findings that VMH^BDNF^ neurons coexpress MC4R and NPY5R and integrate melanocortin and NPY feeding signals^28^. This is also consistent with previous studies^28^ by Xu et al. who reported that BDNF expression in the VMH was increased by melanocortin agonists and that intracerebroventricular BDNF injections ameliorated the hyperphagic phenotype of A^Y^ mice. Although leptin increases both melanocortin signalling and VMH^BDNF^ activity, other leptin-independent signals are also likely to contribute, because ablating these neurons in leptin-sensitive (*ob*/*ob*) and leptin-resistant (DIO) mice increased weight. The data presented here are thus consistent with the possibility that both leptin-dependent and leptin-independent signals regulate the activity of VMH^BDNF^ neurons to establish a set point for weight in lean and obese mice. A leptin-independent effect is also consistent with a previous report showing that *ob*/*ob* mice receiving a 6 month infusion of leptin while on a HFD ended up at a similar weight to wild-type animals fed the same diet^47^. The fact that leptin levels were fixed at a constant low level suggests that another signal besides leptin was limiting the weight of this group. Another study also suggested the existence of signals in addition to leptin^48^. It will now be important to identify the putative leptin-independent signals.

Also consistent with previous reports^10^, our findings suggest that ablation of VMH^BDNF^ neurons accounts for the massive obesity associated with lesions of the VMH in rodents^49^ and humans^50,51^. This effect is not recapitulated by knockout of SF1, a canonical marker expressed in most VMH neurons, or by knockout of BDNF in SF1 neurons^13,52^, suggesting that the VMH^BDNF^ neurons are distinct from SF1-expressing neurons there. We found only limited overlap between BDNF and SF1 neurons, which marked nearly all of the other neurons in this nucleus (around 6% of SF1 neurons also express BDNF according to single-cell RNA sequencing)^16^. Thus, our data and those of others^10^ suggest that the subpopulation of BDNF neurons in VMH accounts for the hyperphagia and massive obesity that follows a VMH lesion. In aggregate, these findings and those of others strongly suggest that VMH^BDNF^ neurons contribute significantly to the obesity associated with BDNF and TrkB mutations. Further support for this conclusion was provided by a recent study that found an interaction of BDNF and astrocytes in the VMH that was essential for the suppression of weight^53^. However, these results do not exclude the possibility that other sites might also contribute to the obesity associated with a BDNF or TrkB mutation. For example, although they do not regulate feeding, BDNF-expressing neurons in the paraventricular nucleus have been shown to regulate energy expenditure, with effects on thermogenesis and adipose tissue innervation^54,55^.

In summary, these data show that VMH^BDNF^ neurons are a key element of a simple circuit that regulates food intake and body weight by directly connecting interoceptive neurons to motor outputs controlling consummatory behaviours and jaw movements. These results provide a framework for studying the role of this Arc → VMH^BDNF^ → pMe5 circuit and factors that modulate it to control feeding.

## Methods

### Mice

All animal care and experimental procedures were ethically performed and approved by the Institutional Animal Care and Use Committee at Rockefeller University. Male mice were single-housed with a 12 h light/12 h dark cycle and ad libitum access to regular chow and water, except in fasting and DIO studies, where either a HFD with 45 kcal%fat (4.7 kcal g^−1^) or a HPD with 42 kcal%fat and high sucrose content (4.5 kcal g^−1^) (TD.88137, Envigo) was provided. We used male *ob*/*ob* (B6.Cg-Lep^ob^/J; 000632, Jackson Laboratory; or bred in-house), Rosa26^fsTRAP^ (B6.129S4-Gt(ROSA)26Sor^tm1(CAG-EGFP/Rpl10a,-birA)Wtp^/J; 022367, Jackson Laboratory), AgRP–Cre (AgRP^tm1(cre)Lowl^/J; 012899, Jackson Laboratory) and Flp reporter mice (RCF-tdTomato, B6.Cg-Gt(ROSA)26Sortm65.2(CAG-tdTomato)Hze/J; 032864, Jackson Laboratory) crossed to Cre reporter mice (Rosa26^fsTRAP^). BDNF–IRES–Cre mice^57^ were provided by W. Shen (Shanghai Institute of Technology). POMC Dre mice were provided by J. Bruning (Max Planck Institute for Metabolism Research). For retrograde tracing from motor neurons in Mo5, Chat–Cre mice (B6.129S-Chattm1(cre)Lowl/MwarJ; 031661, Jackson Laboratory) were crossed to Helper RabV mice (B6;129P2-Gt(ROSA)26Sortm1(CAG-RABVgp4,-TVA)Arenk/J; 024708, Jackson Laboratory) and TrkBF616A knock-in mice carrying a point mutation that renders the receptor sensitive to an allele-specific kinase inhibitor (1-NM PP)^58^. All mouse lines were in a WT (C57BL/6 J) background. For brain surgeries, male mice of at least 8 weeks of age were anesthetized with isoflurane and placed in a stereotaxic frame (David Kopf Instruments), a craniotomy was performed, and a borosilicate glass pipette was used to inject viral vectors. For VMH injections: three injections (each 50 nl) were made into each hemisphere (bregma, −1.36 mm; midline, ±0.35 mm; from brain surface, 5.70 mm, 5.60 mm and 5.50 mm). For injections into the Arc, 50 nl was injected as follows: bregma, −1.45 mm; midline, ±0.45 mm; from brain surface, 5.70 mm, 5.60 mm and 5.50 mm. For injections into Mo5, 75 nl was injected as follows: bregma, −5.20 mm, midline, ±1.5 mm; from brain surface, 4.60 mm and 4.50 mm. For Me5 injections: from bregma, −5.4 mm; midline, ±0.9 mm; from brain surface, 4.5 mm, 4.0 mm, 3.5 mm.

### Reagents

Leptin was diluted in sterile saline (3 mg kg^−1^) and injected intraperitoneally. For photometry recording, mice were fasted overnight and injected with either saline or leptin 2 h before recordings. All mice received saline and leptin injections in alternating order. For acute food intake experiments, leptin or saline was injected 2 h before onset of the dark period, and all mice received saline and leptin injections in a cross-over design.

### Viruses

Cre-dependent neuronal ablation was performed by injection of AAV1-mCherry-flex-dtA (UNC Vector Core)^59^. To target expression of calcium activity indicator GCaMP6s to VMH^BDNF^ neurons, we used an AAV vector carrying a double-floxed GCaMP6s construct (AAV5-Syn-Flex-GCaMP6s-WPRE-SV40, Addgene)^60^. For optogenetic manipulations, a somatic targeting GtACR (AAV5-hSyn1-SIO-stGtACR1-FusionRed)^61^ or ChR (AAV5-EF1a-double-floxed-hChR2(H134R)-EYFP-WPRE-HGHpA)^62^ was used (both Addgene). For long-term silencing, a Cre-dependent TelC AAV (AAV5-hSyn-FLEX-TeLC-P2A-dTomato, Addgene) was used, and for long-term activation a Cre-dependent NaChBac (AAV-Syn-DIO-NaChBac-dTomato) and a Dre-dependent NaChBac^63^ (AAV5-hSyn-roxSTOProx-NaChBac-dTomato, HHMI-Janelia Research Campus) were used. For retrograde tracing, a combination of two helper AAVs (AAV1-TREtight-mTagBFP2-B19G and AAV1-syn-FLEX-splitTVA-EGFP-tTA, both Addgene) and pseudotyped Rabies (EnvA G-Deleted Rabies-mCherry, Salk Institute)^64,65^ were injected; for retrograde labelling from Mo5, a G-deleted Rabies-H2B-mCherry (Salk viral core) was used; and for anatomical tracing from Me5, a retrograde mCherry construct (pAAV-hSyn-DIO-hM4D(Gi)-mCherry, Addgene) was used. For projection activation, we used an AAV encoding eOPN3 (AAV-hSyn1-SIO-eOPN3-mScarlet-WPRE, Addgene)^61^, and for labelling projections with ChR we used a retro-AAV (AAV-EF1a-double-floxed-hChR2(H134R)-mCherry-WPRE-HGHpA). Anterograde labelling was done with AAV1 (ref. ^66^) encoding Cre (AAV-hSyn-Cre-WPRE-hGH, Addgene) and Flp (AAV-EF1a-Flpo, Addgene). For ‘Cre-out’ experiments, AAV-Ef1a-DO-ChETA-EYFP-WPRE-pA (Addgene) was used.

### Immunofluorescence

For c-Fos staining after DIO, BDNF–Cre mice were crossed to Rosa26fsTRAP to express eGFP in a Cre-dependent manner in BDNF neurons. Mice were fed a HFD, while littermate control mice were fed chow. After 16 weeks, mice were transcardially perfused with 4% paraformaldehyde, and their brains were postfixed for 1 day in 4% paraformaldehyde. Brains were then placed in 30% sucrose in phosphate-buffered saline (PBS) until precipitation and frozen and coated in OCT for cryosectioning. Cryosections (50 μm) were cut using a Leica cryostat (CM1950). Brain sections were washed in PBS with 0.1% Triton X-100 (PBST, pH 7.4) and blocked in 3% normal goat/donkey serum (Jackson ImmunoResearch Laboratories) and 2% BSA (Sigma) in PBST for 2 h. Slides were then incubated overnight at room temperature with primary antibody. After being washed in PBST, sections were incubated with fluorescein-conjugated goat IgG. The primary antibodies used and their dilutions were as follows: rabbit anti-FOS (1:1,000; mAb 2250S, Cell Signaling), chicken anti-GFP (1:1,000, ab13970, Abcam). Secondary antibodies conjugated with Alexa-594 and Alexa-488 were purchased from Invitrogen. Brain sections were mounted on to SuperFrost (Fisher Scientific 22-034- 980) slides and then visualized with an inverted Zeiss LSM 780 laser scanning confocal microscope with a ×10 or ×20 lens. Images were imported to Fiji for further analysis and to count cells. To quantify numbers of stained cells, brain slides were imaged under a ×20 objective. For advillin staining, the procedure was the same as above but with rabbit anti-advillin (1:500, NBP2-92263, Novus Biologicals) as the primary antibody and Alexa 647 donkey anti-rabbit (1:500, ab150075, Abcam) as the secondary antibody. For anterograde tracing, brains were processed as described above; tdtomato/Ruby and GFP were amplified with rabbit anti-RFP (1:1000, 600-401-379, Rockland) and chicken anti-GFP (1:1,000, ab13970, Abcam) as primary antibodies, and secondary antibodies conjugated with Alexa-594 and Alexa-488 were purchased from Invitrogen.

### In situ hybridization

Mice were briefly transcardially perfused with RNase-free PBS to remove blood. Brains were then quickly collected, frozen in OCT and stored at −80 °C until they were sectioned by cryostat (15 μm sections) and attached on Superfrost Plus Adhesion Slides (Thermo Fisher). RNAscope Fluorescent Multiplex assay (Advanced Cell Diagnostics Bio) was then performed using the RNAscope system as per the manufacturer’s protocol. Probes for the following mRNAs were used (all from ACDBio): mm-BDNF (catalogue no. 424821) and eGFP (catalogue no. 400281), VGlut2 (catalogue no. 319171), RabV (catalogue no. 456781), AgRP (catalogue no. 400711), POMC (catalogue no. 314081), MC4R (catalogue no. 319181-C2) and NPY5R (catalogue no. 589811), LepR (catalogue no. 402731). Briefly, a hydrophobic barrier was created using Immedge Hydrophobic Barrier Pen (Vector Laboratories). Slides were pretreated by serial submersion in 1× PBS, 50% EtOH, 70% EtOH and twice 100% EtOH for 2 min each, at room temperature. Probe hybridization was achieved by incubation of 35 μl mRNA target probes for 2 h at 40 °C using a HyBez oven. The signal was amplified by subsequent incubation of Amp-1, Amp-2, Amp-3 and Amp-4, one drop each, for 30, 15, 30 and 15 min, respectively, at 40 °C using a HyBez oven. Each incubation step was followed by two 2 min washes with RNAscope washing buffer. Nucleic acids were stained using DAPI Fluoromount-G (SouthernBiotech) mounting medium before coverslipping. Slides were visualized with an inverted Zeiss LSM 780 laser scanning confocal microscope using a ×20 or ×40 lens. Images were imported to Fiji for further analysis.

### Long-term body weight and food intake measures

Single-housed mice were measured weekly to assess body weight and food intake. Whole-body composition was measured using nuclear magnetic resonance relaxometry (EchoMRI) at the end of the 16 week period.

### Optogenetics

After injection of AAVs encoding either ChR or GtACR, we bilaterally implanted 200 μm fibre optic cannulas (Thorlabs) in BDNF–Cre mice and control mice (Cre-negative littermates). For VMH targeting, implants were angled at 15° and placed at the following positions: bregma, −1.36 mm; midline, ±1.85 mm; from brain surface: 5.25 mm. For brainstem targeting, implants were angled at 15° and placed at the following positions: bregma, −5.4 mm; midline, ±1.75 mm; from brain surface, 2.6 mm (for Me5); bregma, −6.3 mm; midline, ±2.15 mm; from brain surface, 5.5 mm (for LPGi); and bregma, −5.7 mm; midline, ±2.6 mm; from brain surface, 4.7 mm (for PCRT). Implants were subsequently fixed with dental cement (C&B Metabond). After a minimum of 3 weeks expression time, mice were handled and habituated to tethering with optical fibres. A constant 473 nm laser (OEM Lasers/OptoEngine) was used for optogenetic inhibition with GtACR and pulsed at 2 Hz (5 ms) for optoactivation with ChR. For inhibition with OPN, a 532 nm laser at 10 Hz was used. Lasers were connected to bifurcated optical fibres (Thorlabs) with an output of approximately 2–5 mW at the implant. For AgRP projection stimulation, the laser power was reduced to 1–2 mW. Food intake studies were done in home cage-like arenas during the light phase without bedding unless otherwise stated.

#### Acute food intake experiments

With optogenetic activation or inhibition, mice were habituated to the arena for 10 min without food present. Then, consumption of a single food pellet was measured every 30 min for five repetitions, with only the second repetition being paired with optogenetic activation or inhibition. For open loop and closed loop feeding experiments, a single chow pellet was fixed to the middle of a home cage-style arena with fun-tak (Loctite). Food intake was assessed every 5 min in three repetitions, with only the second repetition being paired with optogenetic inhibition. Inhibition was either 5 min constant laser (open loop) or triggered (closed loop) by real-time video tracking (Noldus, Ethovision) whenever the head of the mouse was within a 3 cm radius of the pellet. For modification with bedding present, the same open loop set-up was used but with corn cob bedding covering the floor. For wood block trials, the chow pellet was replaced by a wood block that was fixed with fun-tak. Time spent biting the wood block was manually assessed and quantified by scoring of video recordings.

#### Liquid diet experiments

Ensure Vanilla (20 µl) was pipetted on to the bare floor of a cage in three repetitions without light activation, followed by three repetitions with light activation and another three repetitions without light activation. For quantification purposes, experiments were video recorded, and latencies from Ensure delivery to full consumption were scored and averaged over the three repetitions.

#### Operant conditioning

Trials were performed in a home cage-style arena with two capacitive touch plates mounted on opposite sides. Both touch plates were connected to an Arduino to register numbers of touches, and one randomly assigned side would trigger cessation of a constant 2 Hz laser (for ChR) for 3 s or activation of a constant laser for 3 s (for GtACR). Trials lasted for 1 h.

#### Conditioned flavour preference assays

Assays were performed as previously described^44^. Briefly, mice were habituated overnight to orange- and strawberry-flavoured sugar-free Juicy Gels (Hunt’s). Initial preference was assessed in a 30 min session without any light application. The preferred flavour was then paired with light exposure for ChR mice, or the less preferred flavour paired with light exposure for GtACR mice. Conditioning was repeated daily for 3 days and consisted of one light exposure session in which light exposure started after 5 min and lasted for 25 min while the paired gel was presented and a 30 min session with the non-paired gel without any light exposure. A 15 min test session in which both gels were available was performed on the day after conditioning ended.

#### Spaghetti and wooden stick experiments

Five spaghetti sticks or wooden sticks of similar length were distributed equally in an empty home cage. Control and eOPN3 mice were given 5 min baseline exploration time, 5 min with laser inhibition and 5 min without laser with the spaghetti or sticks present. A side and overhead camera were used to quantify the time spent chewing.

### Head-fixed jaw-tracking

Mice for head-fixed experiments had a small metal bar fixed to their skull with dental cements during implant surgery. After a minimum of 3 weeks recovery, mice were habituated to being head-fixed in a custom head-fixation set-up. This set-up consisted of a side camera (Basler a2A1920-160umPRO -ace 2) and a laser source controlled and synchronized by Bonsai^67^. Frames were acquired at 100 Hz at 722 × 878 pixel size. Optogenetic inhibition trials consisted of 1 min with laser, 1 min on and 1 min off. Jaw pose was subsequently estimated with DeepLabCut^68^.

### Fibre photometry

After injection of an AAV encoding Cre-dependent GCaMP6s into the VMH of male BDNF–Cre mice, a unilateral 400 μm fibre optic cannula was implanted as described for optogenetics. After a minimum of 4 weeks expression time, mice were habituated to tethering and a home cage-style arena.

Data were collected with a Fiber Photometry system by Tucker-Davis Technologies (RZ5P, Synapse), and Doric components and recordings were synched to video recordings in Ethovision by TTL triggering. A 465 nm and isosbestic 405 nm LED (Doric) were reflected into a dual fluorescence Mini Cube (Doric) before entering the recording fibre that connects to the implant. Recording fibres were photobleached overnight before recordings to minimize autofluorescence. GCaMP6s fluorescence was collected as a calcium-dependent signal (525 nm) and isosbestic control (430 nm) with a femtowatt photoreceiver (Newport, 2151) and a lock-in amplifier using the RZ5P at a 1 kHz sampling rate.

Mice were allowed to habituate for 30 min at the start of each recording session before any items were introduced into the arena. Feeding bouts were manually assessed and scored from video recordings when mice were given single pellets of chow or 20 mg sucrose treat pellets (Bio-Serv). Instances of food interaction without consumption were defined as approach within a 2 cm radius around a chow pellet without subsequent consumption. To measure the effects of different energy states, the same mice underwent the same standardized recording procedure in the following states: lean ad lib chow-fed, overnight fasted injected with saline, and overnight fasted injected with leptin and 4 weeks DIO. The order of the lean, fasted saline and fasted leptin states was randomized to avoid any order effects.

A script written in MATLAB based on a previously published method and code was used for analysis^69^. Bleaching and movement artefacts were removed by applying a polynomial least-squares fit to the 405 nm signal, adjusting it to the 465 nm trace (405_fitted_) to then calculate the GCaMP signal as %Δ*F*/*F* = (465_signal_ − 405_fitted_)/405_fitted_. Traces were filtered with a moving average filter and downsampled by a factor of 20. Three trials per mouse were averaged to derive data for peri-event plots and analysis of maximum and minimum signals.

### Quantification and statistics

Sample sizes were chosen on the basis of similar studies previously published and kept to a minimum to reduce unnecessary use of animals. Experimenters were blinded to group allocation as much as possible, but small groups sizes and concurrent recordings of control and treatment animals meant it was sometimes not possible. Group allocation was done at random, unless genetic backgrounds dictated group assignment. Microscopy images were analysed and quantified in ImageJ/Fiji. Photometry recordings were processed and analysed with MATLAB (MathWorks). Statistical analyses were performed in GraphPad Prism. All tests were two-sided, and results are displayed as mean ± s.e.m. Statistical details are provided in the figure legends and source data, including definitions of *n* and significance. Significance was defined as *P* < 0.05. Mice were randomized into control or treatment groups. Control mice were age-matched littermate controls where possible. Graphs were produced using GraphPad Prism and Adobe Illustrator, and schematic illustrations were prepared in BioRender.

### Reporting summary

Further information on research design is available in the Nature Portfolio Reporting Summary linked to this article.

## Online content

Any methods, additional references, Nature Portfolio reporting summaries, source data, extended data, supplementary information, acknowledgements, peer review information; details of author contributions and competing interests; and statements of data and code availability are available at 10.1038/s41586-024-08098-1.

## Supplementary information

Reporting Summary

Supplementary Video 1 Inhibition of VMH^BDNF^ neurons causes eating-like behaviour targeted at a wood block: Mice biting a wood block in response to optogenetic inhibiting of VMH^BDNF^ neurons when a mouse is near a wood block.

Supplementary Video 2 Photometry recording of VMH^BDNF^ neurons during food consumption: photometry recording of a mouse approaching and consuming a treat pellet.

Supplementary Video 3 Photometry recording of VMH^BDNF^ neurons during food rejection: photometry recording of a mouse approaching and not consuming a chow pellet.

Supplementary Video 4 Mouse eating spaghetti without laser: representative recording of OPN mouse interacting with spaghetti without laser (4× speed).

Supplementary Video 5 Inhibition of VMH^BDNF^ to pMe5 projections causes spaghetti biting: representative recording of OPN mouse interacting with spaghetti during VMH^BDNF^ to Me5 inhibition (4× speed).

Supplementary Video 6 Inhibition of VMH^BDNF^ to pMe5 projections causes wood-stick biting: representative recording of OPN mice interacting with wooden sticks during VMH^BDNF^ to Me5 inhibition (4× speed).

Supplementary Video 7 Jaw movements and tracking during inhibition of VMH^BDNF^ neuron-to-Me5 projections (5× speed): synchronized video of jaw movements before, during and after laser silencing of VMH^BDNF^ neuron projections to pMe5 with position of jaw in *x*-*y* space.

Supplementary Video 8 Jaw movements and tracking in a control mouse (5× speed): synchronized video of jaw movements before, during and after laser-on of a control mouse with position of jaw in *x*-*y* space.

## Source data

Source Data Figs. 1–6

Source Data Extended Data Figs. 1–6

## Data Availability

All data generated or analysed during this study are included in the article and its Supplementary Information files. Source data are provided with this paper.

## Code availability

The code for photometry analysis, Arduino code for operant conditioning, and Bonsai code for head-fixed behaviour and analysis are available from GitHub (https://github.com/ckosse/Jaw_2024).
