# Supplementary material for: A subcortical feeding circuit linking an interoceptive node to jaw movement
Source: Nature. 2024 Oct 23;636(8041):151–61. doi: 10.1038/s41586-024-08098-1 (PMC11618074; doi:10.1038/s41586-024-08098-1)
Supplement: Supplementary file 1 — Reporting Summary [file 41586_2024_8098_MOESM1_ESM.pdf]

nature portfolio

Corresponding author(s): Jeffrey Friedman

Last updated by author(s): 8/16/2024

Reporting Summary

Nature Portfolio wishes to improve the reproducibility of the work that we publish. This form provides structure for consistency and transparency in reporting. For further information on Nature Portfolio policies, see our Editorial Policies and the Editorial Policy Checklist.

Statistics

For all statistical analyses, confirm that the following items are present in the figure legend, table legend, main text, or Methods section.

n/a	Confirmed	
☐	☒	The exact sample size (*n*) for each experimental group/condition, given as a discrete number and unit of measurement
☐	☒	A statement on whether measurements were taken from distinct samples or whether the same sample was measured repeatedly
☐	☒	The statistical test(s) used AND whether they are one- or two-sided<br>*Only common tests should be described solely by name; describe more complex techniques in the Methods section.*
☒	☐	A description of all covariates tested
☐	☒	A description of any assumptions or corrections, such as tests of normality and adjustment for multiple comparisons
☐	☒	A full description of the statistical parameters including central tendency (e.g. means) or other basic estimates (e.g. regression coefficient) AND variation (e.g. standard deviation) or associated estimates of uncertainty (e.g. confidence intervals)
☐	☒	For null hypothesis testing, the test statistic (e.g. *F*, *t*, *r*) with confidence intervals, effect sizes, degrees of freedom and *P* value noted<br>*Give P values as exact values whenever suitable.*
☒	☐	For Bayesian analysis, information on the choice of priors and Markov chain Monte Carlo settings
☒	☐	For hierarchical and complex designs, identification of the appropriate level for tests and full reporting of outcomes
☒	☐	Estimates of effect sizes (e.g. Cohen's *d*, Pearson's *r*), indicating how they were calculated

Our web collection on statistics for biologists contains articles on many of the points above.

Software and code

Policy information about availability of computer code

Data collection	Behavior tracking was done with Ethovision XT (version 9.0).<br>Fiber photometry data was acquired using TDT synapse software.<br>Touches during the self-inhibition experiments were collected with a Arduino code (version 1.8.9).
Data analysis	Photometry data was processed using a custom Matlab code (version R2019a). Image analysis for in situ hybridization and immunhistochemistry was done in Fiji ImageJ (version 1.53c). All other data was analyzed using GraphPad Prism (version 9.0.0)

For manuscripts utilizing custom algorithms or software that are central to the research but not yet described in published literature, software must be made available to editors and reviewers. We strongly encourage code deposition in a community repository (e.g. GitHub). See the Nature Portfolio guidelines for submitting code & software for further information.

Data

Policy information about availability of data

All manuscripts must include a data availability statement. This statement should provide the following information, where applicable:

- Accession codes, unique identifiers, or web links for publicly available datasets
- A description of any restrictions on data availability
- For clinical datasets or third party data, please ensure that the statement adheres to our policy

Data availability
All data generated or analyzed during this study are included in the article and its supplementary information files. Source data are provided with this paper.

Research involving human participants, their data, or biological material

Policy information about studies with human participants or human data. See also policy information about sex, gender (identity/presentation), and sexual orientation and race, ethnicity and racism.

Reporting on sex and gender	*Use the terms sex (biological attribute) and gender (shaped by social and cultural circumstances) carefully in order to avoid confusing both terms. Indicate if findings apply to only one sex or gender; describe whether sex and gender were considered in study design; whether sex and/or gender was determined based on self-reporting or assigned and methods used. Provide in the source data disaggregated sex and gender data, where this information has been collected, and if consent has been obtained for sharing of individual-level data; provide overall numbers in this Reporting Summary. Please state if this information has not been collected. Report sex- and gender-based analyses where performed, justify reasons for lack of sex- and gender-based analysis.*
Reporting on race, ethnicity, or other socially relevant groupings	*Please specify the socially constructed or socially relevant categorization variable(s) used in your manuscript and explain why they were used. Please note that such variables should not be used as proxies for other socially constructed/relevant variables (for example, race or ethnicity should not be used as a proxy for socioeconomic status). Provide clear definitions of the relevant terms used, how they were provided (by the participants/respondents, the researchers, or third parties), and the method(s) used to classify people into the different categories (e.g. self-report, census or administrative data, social media data, etc.) Please provide details about how you controlled for confounding variables in your analyses.*
Population characteristics	*Describe the covariate-relevant population characteristics of the human research participants (e.g. age, genotypic information, past and current diagnosis and treatment categories). If you filled out the behavioural & social sciences study design questions and have nothing to add here, write "See above."*
Recruitment	*Describe how participants were recruited. Outline any potential self-selection bias or other biases that may be present and how these are likely to impact results.*
Ethics oversight	*Identify the organization(s) that approved the study protocol.*

Note that full information on the approval of the study protocol must also be provided in the manuscript.

Field-specific reporting

Please select the one below that is the best fit for your research. If you are not sure, read the appropriate sections before making your selection.

☒ Life sciences ☐ Behavioural & social sciences ☐ Ecological, evolutionary & environmental sciences

For a reference copy of the document with all sections, see nature.com/documents/nr-reporting-summary-flat.pdf

Life sciences study design

All studies must disclose on these points even when the disclosure is negative.

Sample size	Sample sizes were chosen based on similar studies previously published and kept at a minimum to reduce unnecessary usage of animals.
Data exclusions	Preestablished criteria was used for inclusion/exclusion of data from studies. Subjects with mistargeted viral injections or aberrant implants were excluded from analyses
Replication	Experiments were performed across multiple animal replicates, as denoted in each experiment.
Randomization	Mice were randomly assigned when possible to control and experimental groups.
Blinding	Experimenters were blinded to group allocation as much as possible but small groups sizes and concurrent recordings of control and treatment animals made it sometimes not possible

Reporting for specific materials, systems and methods

We require information from authors about some types of materials, experimental systems and methods used in many studies. Here, indicate whether each material, system or method listed is relevant to your study. If you are not sure if a list item applies to your research, read the appropriate section before selecting a response.

Materials & experimental systems

n/a	Involved in the study
☐	☒ Antibodies
☒	☐ Eukaryotic cell lines
☒	☐ Palaeontology and archaeology
☐	☒ Animals and other organisms
☒	☐ Clinical data
☒	☐ Dual use research of concern
☒	☐ Plants

Methods

n/a	Involved in the study
☒	☐ ChIP-seq
☒	☐ Flow cytometry
☒	☐ MRI-based neuroimaging

Antibodies

Antibodies used	rabbit anti-FOS (1:1,000; mAb 2250S, Cell Signaling), chicken anti-GFP (1:1,000, ab 13970, Abcam), anti-RFP (1:1000, 600-401-379, Rockland)
Validation	GFP1020 - PMIDs: 31372393,30850733

Animals and other research organisms

Policy information about studies involving animals; ARRIVE guidelines recommended for reporting animal research, and Sex and Gender in Research

Laboratory animals	male mice from 3 to 12 months of age. ob/ob (B6.Cg-Lepob/J; 000632, Jackson Laboratory; or bred in-house), Rosa26fsTRAP (B6.129S4-Gt(ROSA)26Sortm1(CAG-EGFP/Rpl10a,-birA)Wtp/J; 022367, Jackson Laboratory), AgRP-cre (AgRPtm1(cre)Lowl/J; 012899, Jackson Laboratory), Flp reporter mice (RCF-tdTomato, B6.Cg-Gt(ROSA)26Sortm65.2(CAG-tdTomato)Hze/J; 032864, Jackson Laboratory) crossed to cre reporter mice (Rosa26fsTRAP). BDNF-IRES-Cre mice were provided by W. Shen (Shanghai Institute of Technology). POMC-dre were provided by J. Bruning (Max Planck Institute for Metabolism Research). All mouse lines are in a WT (C57BL/6J) background.
Wild animals	NA
Reporting on sex	Only male mice were used
Field-collected samples	NA
Ethics oversight	all animal experiments were approved by the Rockefeller University IACUC, according to NIH guidelines

Note that full information on the approval of the study protocol must also be provided in the manuscript.

Plants

Seed stocks	NA
Novel plant genotypes	NA
Authentication	NA
